# Correction: An *in vitro* method for inducing titan cells reveals novel features of yeast-to-titan switching in the human fungal pathogen *Cryptococcus gattii*

**DOI:** 10.1371/journal.ppat.1011001

**Published:** 2022-11-29

**Authors:** Lamin Saidykhan, Joao Correia, Andrey Romanyuk, Anna F. A. Peacock, Guillaume E. Desanti, Leanne Taylor-Smith, Maria Makarova, Elizabeth R. Ballou, Robin C. May

Fig 8 incorrectly appears without panel B. The authors have provided a corrected version of Fig 8 here.

**Fig 8:**
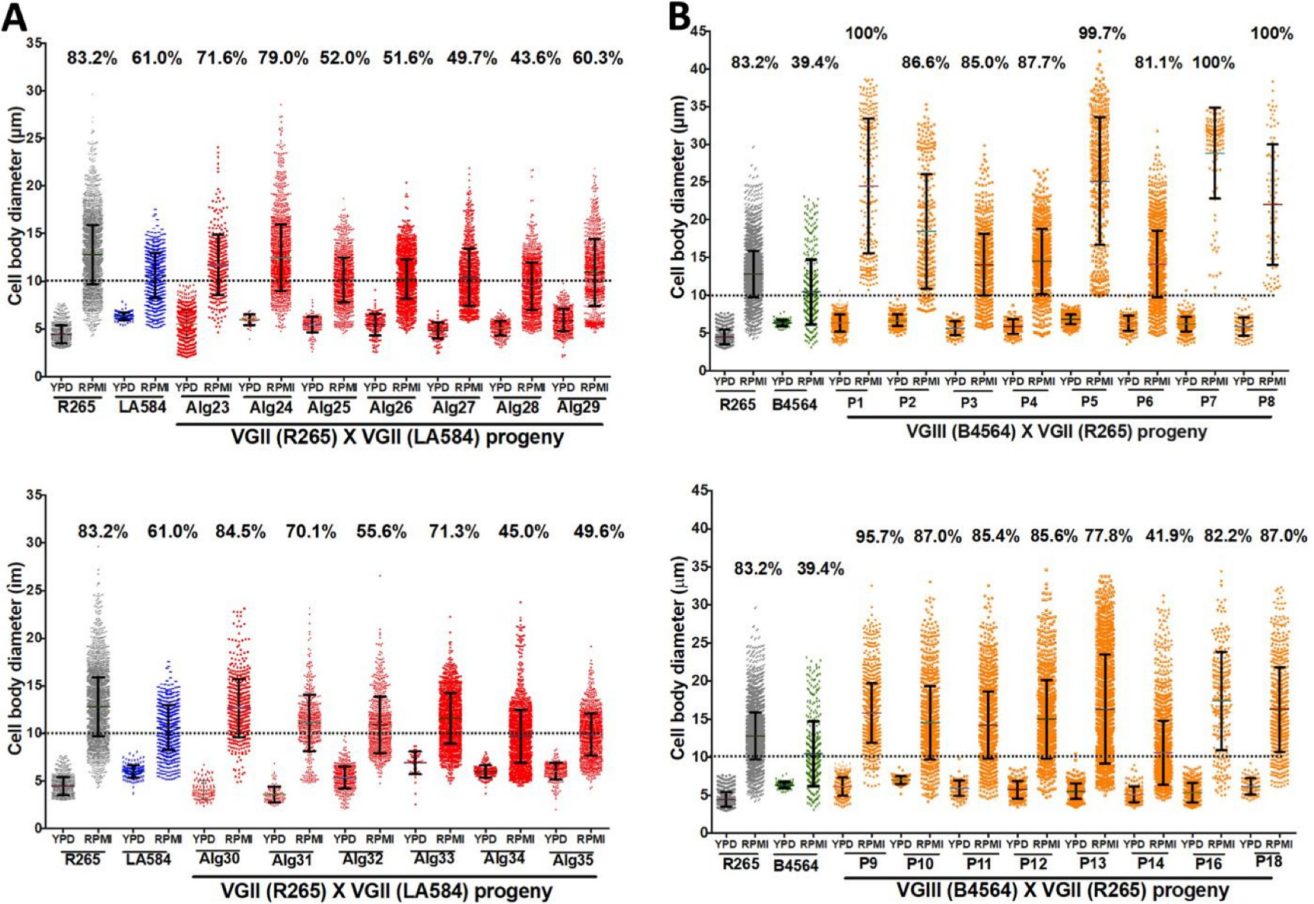
Capacity to form titan cells of *C*. *gattii* progeny arising from two crosses. (A) Titanisation pattern following three days of induction for R265 (VGII) x LA584 (VGII) and 13 progeny (Alg23-Alg35) arising from this cross [33]. (B) Titanisation pattern following three days of induction of R265 (VGII) x B4564 (VGIII) and 18 of the progeny (P1-P18) arising from this cross.
