# Supplementary material for: An in vitro method for inducing titan cells reveals novel features of yeast-to-titan switching in the human fungal pathogen Cryptococcus gattii
Source: PLoS Pathog. 2022 Aug 15;18(8):e1010321. doi: 10.1371/journal.ppat.1010321 (PMC9426920; doi:10.1371/journal.ppat.1010321)
Supplement: S1 Fig — A) Cell body size of R265 yeast cells before (in YPD) and after incubation in sterile RPMI and serum supplemented RPMI. The cells were grown in YPD overnight and in RPMI (sterile and serum-contained) for 3 days in 5% CO2 at 37°C and recovered for cell body size measurement. B) Micrograph of R265 yeast cells after grown in YPD for 7 days in 5%CO2 at 37°C. Scale bar = 15μm. C) pH of YPD amd RPMI before and during titan induction (24 hr and 7 days). Titan cell induction was performed with the R265 (C. gattii) and pH was measured. D) Effect of glucose on titan cell formation. DMEM media was supplemented with D glucose reaching 2000mg/L (the concentration in RPMI) and tested for capacity to induce cell enlargement (>15μm) as compared to RPMI. Titan induction was performed by incubating R265 yeast cells in the different media conditions for 24 hrs at 37°C in 5% CO2. E) The influence of pABA on titan induction in C. neoformans (H99). Cell body size was measured after H99 yeast cells were grown in the different induction media at 37°C in 5% CO2 for 72 hr. (DOCX) [file ppat.1010321.s001.docx]

**S1 Fig:**

**A. Cell body size of R265 yeast cells before (in YPD) and after incubation in sterile RPMI and serum supplemented RPMI.** The cells were grown in YPD overnight and in RPMI (sterile and serum-contained) for 3 days in 5% CO_2_ at 37°C and recovered for cell body size measurement.

**B. Micrograph of R265 yeast cells after grown in YPD for 7 days in 5%CO_2_ at 37°C.** Scale bar=15µm.

**
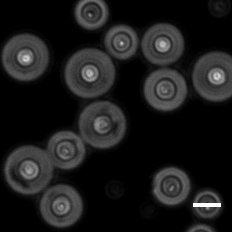
**

**C. pH of YPD amd RPMI before and during titan induction (24 hr and 7 days).** Titan cell induction was performed with the R265 (*C. gattii*) and pH was meausred .

| **Condition** | **YPD (pH)** | **RPMI (pH)** |
| --- | --- | --- |
| Before induction | **6.8** | **8.01** |
| At 24 hr induction | **6.2** | **7.31** |
| At 7 days induction | **7.38** | **7.38** |

**D. Effect of glucose on titan cell formation.** DMEM media was supplemented with D glucose reaching 2000mg/L (the concentration in RPMI) and tested for capacity to induce cell enlargement (>15µm) as compared to RPMI. Titan induction was performed by incubating R265 yeast cells in the different media conditions for 24 hrs at 37°C in 5% CO_2_.


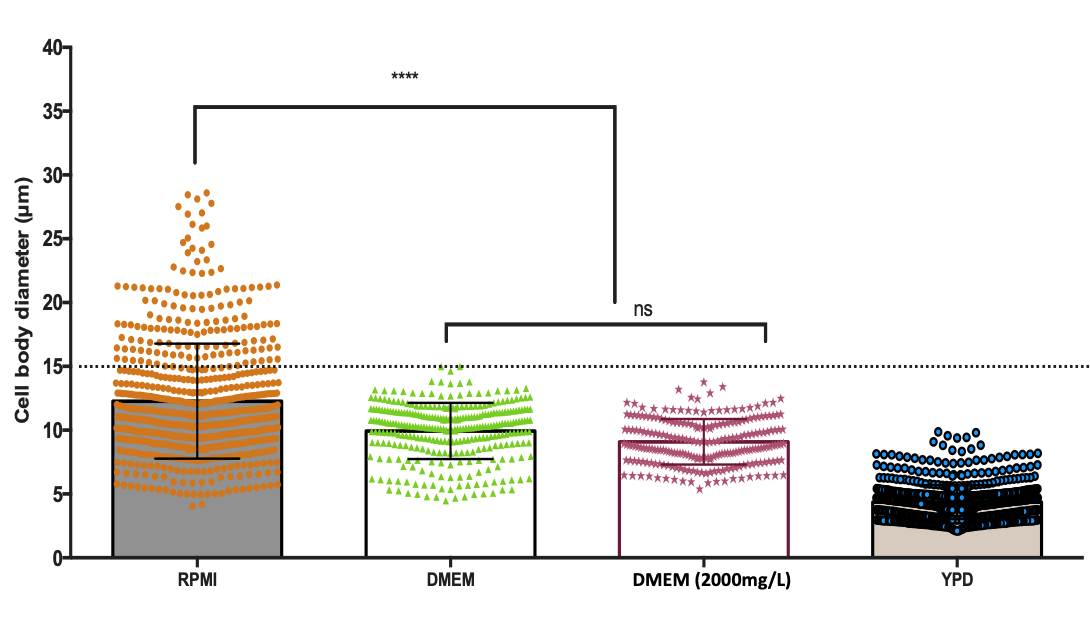


**E. The influence of pABA on titan induction in *C. neoformans* (H99).** Cell body size was measured after H99 yeast cells were grown in the different induction media at 37°C in 5% CO_2_ for 72 hr.
