# Supplementary material for: An in vitro method for inducing titan cells reveals novel features of yeast-to-titan switching in the human fungal pathogen Cryptococcus gattii
Source: PLoS Pathog. 2022 Aug 15;18(8):e1010321. doi: 10.1371/journal.ppat.1010321 (PMC9426920; doi:10.1371/journal.ppat.1010321)
Supplement: S2 Fig — A) Effect of 5% CO2 on cryptococcal growth. Cells incubated at 37°C in serum-free RPMI in 5% CO2 or atmospheric conditions for 24 hrs were assessed for colony forming unit (CFU) on YPD after to evaluate viability. Statistical significance was confirmed by a Two-tailed t-test where ** = p<0.05. B) Proliferation of R265 Titan cells after re-culturing in RPMI. Titan cells obtained from induced cultures after 7 days (A) were re-cultured in fresh, serum-free RPMI at 37°C in 5% CO2 for 24 hrs (B) and analysed microscopically for ability to bud. Scale bar = 15μm. (DOCX) [file ppat.1010321.s002.docx]

**Fig. S2**

**A. Effect of 5% CO_2_** **on cryptococcal growth.** Cells incubated at 37°C in serum-free RPMI in 5% CO_2_ or atmospheric conditions for 24 hrs were assessed for colony forming unit (CFU) on YPD after to evaluate viability. Statistical significance was confirmed by a Two-tailed t-test where **=p<0.05.


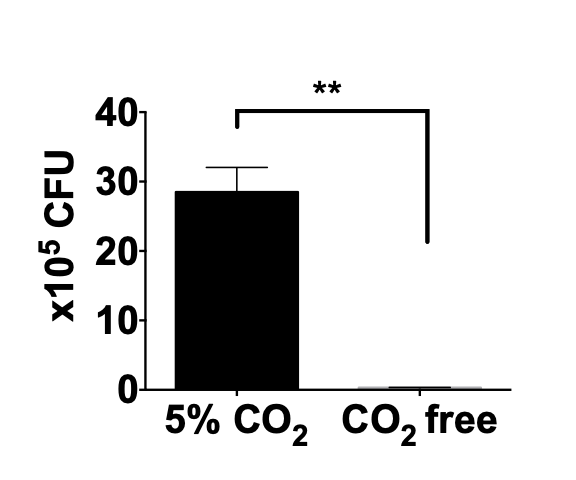


**B. Proliferation of R265 Titan cells after re-culturing in RPMI.** Titan cells obtained from induced cultures after 7 days (A) were re-cultured in fresh, serum-free RPMI at 37°C in 5% CO_2_ for 24 hrs (B) and analysed microscopically for ability to bud. Scale bar= 15µm.


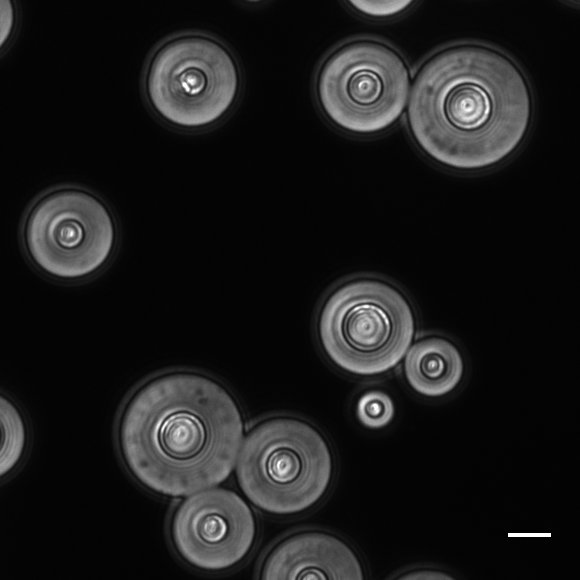

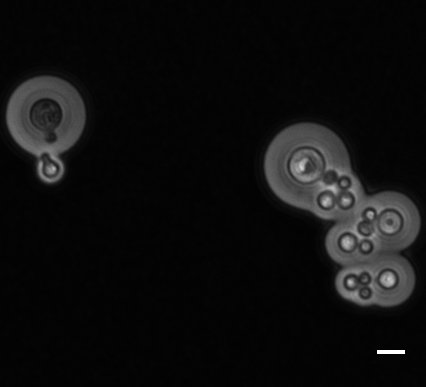


A B
