# Supplementary material for: An in vitro method for inducing titan cells reveals novel features of yeast-to-titan switching in the human fungal pathogen Cryptococcus gattii
Source: PLoS Pathog. 2022 Aug 15;18(8):e1010321. doi: 10.1371/journal.ppat.1010321 (PMC9426920; doi:10.1371/journal.ppat.1010321)
Supplement: S3 Fig — A) Images showing the budding nature of daughter cells before and after titan induction for 24hr, 72 hr and 7 days. Scale bar = 5μm. B) Budding index of daughter cells before and after titanisation. C) Cell body diameter was measured microscopically, and percentage of titan cells determined based on >10μm cell size. The data represents three independent biological repeats and significance was confirmed by one-way ANOVA where, **** = p<0.0001 D) Daughter cells of R265 titan cells were isolated and returned to titan inducing condition and their ploidy was determined by DNA content measurement via flow cytometry before titan induction (green), after 24 hr (blue) and 7 days (brown) relative to R265 haploid yeast (red). E) Flow cytometry data showing the gating strategy employed to confirm cell ploidy of R265 titan daughter cells as compared to YPD grown yeast and (>20μm) filtered titan cells. (DOCX) [file ppat.1010321.s003.docx]

**Fig. S3**

**Characterization of budding nature and titanisation of R265 titan-derived daughter cells**. A) Images showing the budding nature of daughter cells before and after titan induction for 24hr, 72 hr and 7 days. Scale bar= 5µm B) Budding index of daughter cells before and after titanisation. C) Cell body diameter was measured microscopically, and percentage of titan cells determined based on >10µm cell size. The data represents three independent biological repeats and significance was confirmed by one-way ANOVA where, ****=p<0.0001 D) Daughter cells of R265 titan cells were isolated and returned to titan inducing condition and their ploidy was determined by DNA content measurement via flow cytometry before titan induction (green), after 24 hr (blue) and 7 days (brown) relative to R265 haploid yeast (red). E) Flow cytometry data showing the gating strategy employed to confirm cell ploidy of R265 titan daughter cells as compared to YPD grown yeast and (>20µm) filtered titan cells.

**E**

**Gating strategy for YPD grown cells**

**Gating strategy for R265 titan daughter cells**

**Gating strategy for R265 titan (>20µm) cells**
