# Supplementary material for: An in vitro method for inducing titan cells reveals novel features of yeast-to-titan switching in the human fungal pathogen Cryptococcus gattii
Source: PLoS Pathog. 2022 Aug 15;18(8):e1010321. doi: 10.1371/journal.ppat.1010321 (PMC9426920; doi:10.1371/journal.ppat.1010321)
Supplement: S4 Fig — All strains were induced for titan cell formation according to our in vitro protocol. The cell body diameter and ploidy (see S5 Fig) were determined after 72hrs as described in the Methods section. A, B, C, D) Cell body size distribution of isolates from C. gattii complex (VGI-VGIV) before induction (YPD) and after 72 hr of induction (RPMI). E, F) Cell body size distribution of isolates from C. neoformans (VNI-VNII) and C. deneoformans (VNIV) before (YPD) and after 72 hr induction (RPMI) respectively. (DOCX) [file ppat.1010321.s004.docx]

**S4 Figure: Cell body diameter of 42 YPD grown and titan-induced cryptococcal isolates representing the different genotypes within the *C. neoformans/gattii* species complex.**

All strains were induced for titan cell formation according to our *in vitro* protocol. The cell body diameter and ploidy (see S5 Fig.) were determined after 72hrs as described in the “methods” section. A, B, C, D) Cell body size distribution of isolates from *C. gattii* complex (VGI-VGIV) before induction (YPD) and after 72 hr of induction (RPMI). E, F) Cell body size distribution of isolates from *C. neoformans* (VNI-VNII) and *C. deneoformans* (VNIV) before (YPD) and after 72 hr induction (RPMI) respectively.
