# Supplementary material for: An in vitro method for inducing titan cells reveals novel features of yeast-to-titan switching in the human fungal pathogen Cryptococcus gattii
Source: PLoS Pathog. 2022 Aug 15;18(8):e1010321. doi: 10.1371/journal.ppat.1010321 (PMC9426920; doi:10.1371/journal.ppat.1010321)
Supplement: S5 Fig — All isolates were induced for titanisation according our in vitro induction model (as mentioned in the Methods sections) and DNA content was assessed by DAPI staining and flow cytometry analysis. (DOCX) [file ppat.1010321.s005.docx]

**S5 Figure: DNA content of 42 YPD grown (red) and titan-induced (blue) cryptococcal isolates representing the different genotypes within the *C. neoformans/gattii* complex.** All isolates were induced for titanisation according our *in vitro* induction model (as mentioned in the methods sections) and DNA content was assessed by DAPI staining and flow cytometry analysis.

| **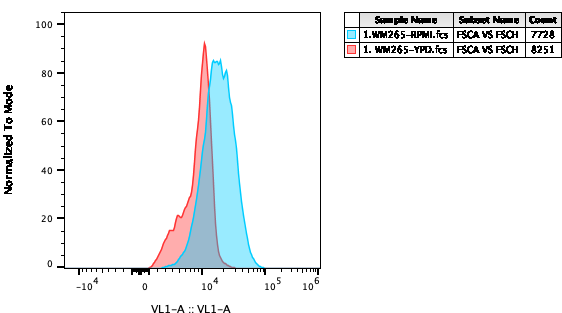** | **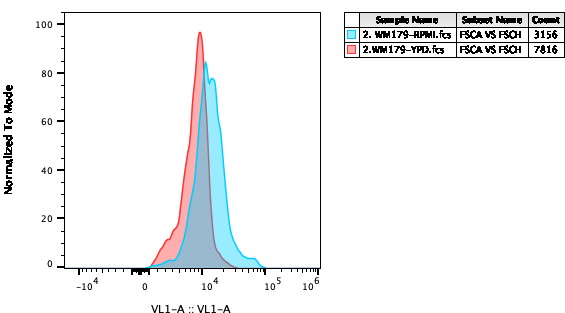** | 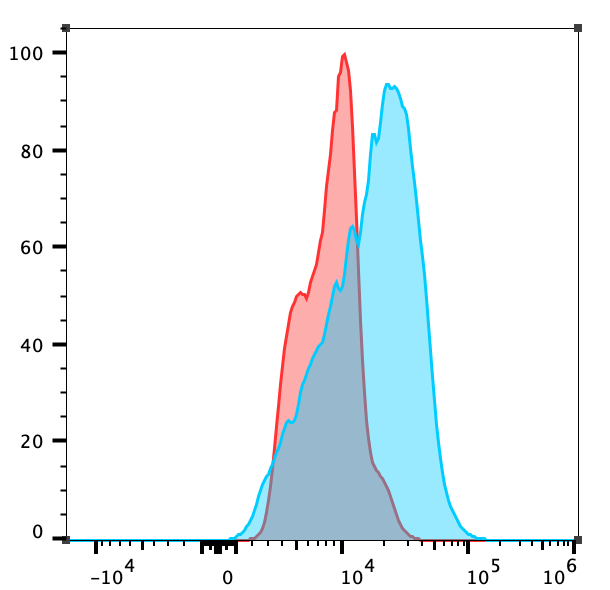 | 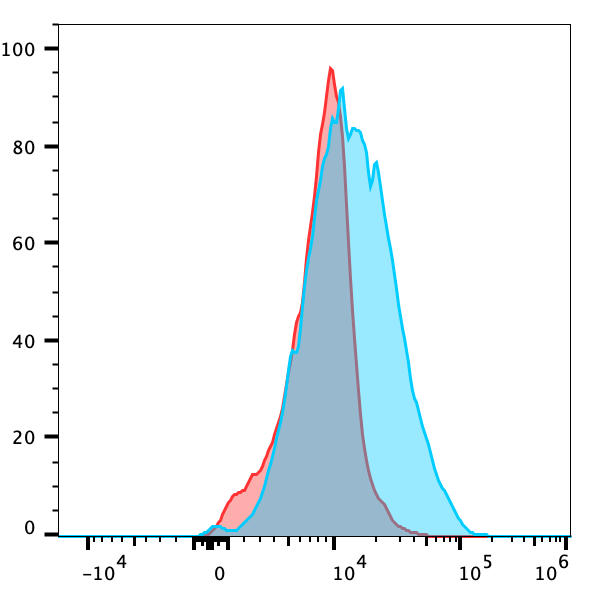 | 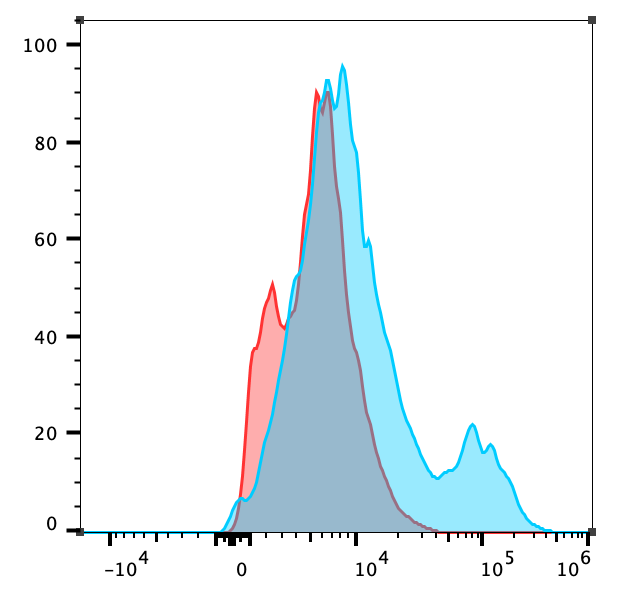 | 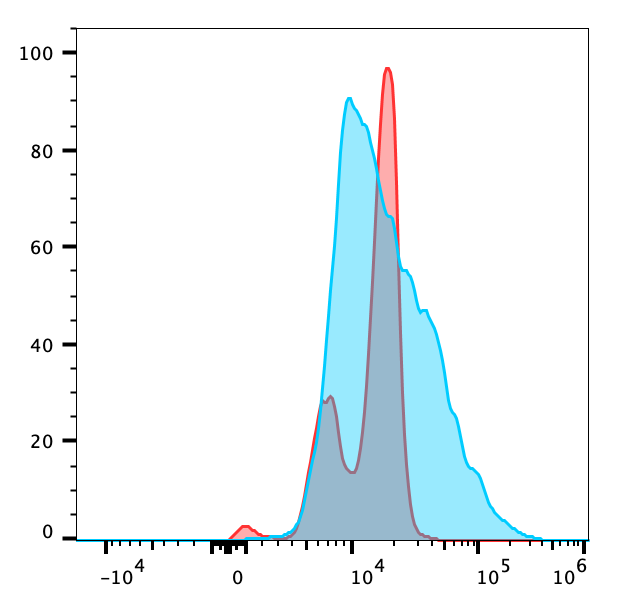 | 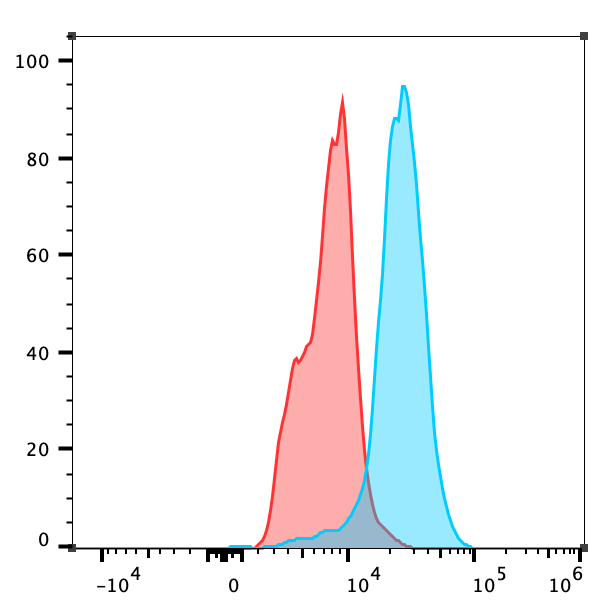 | 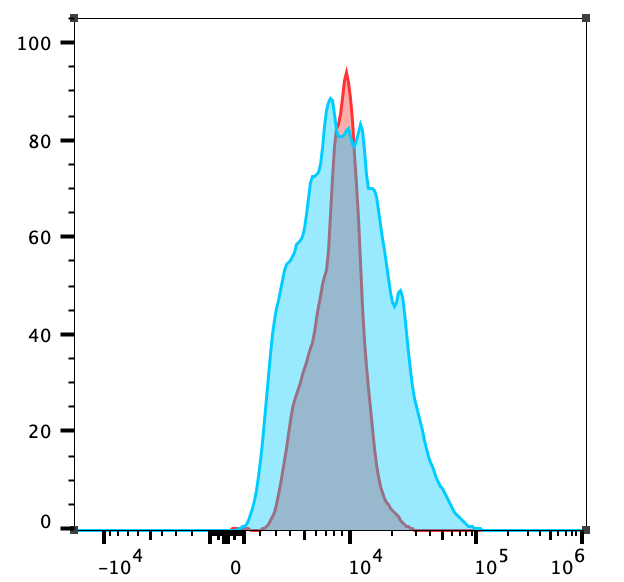 | 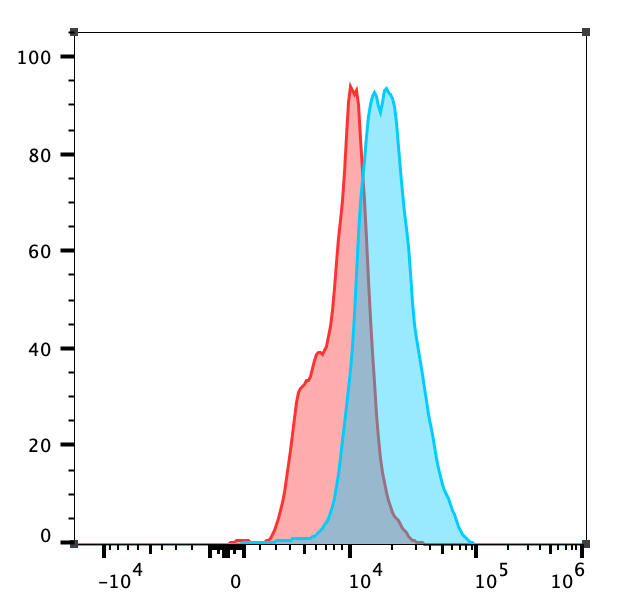 | 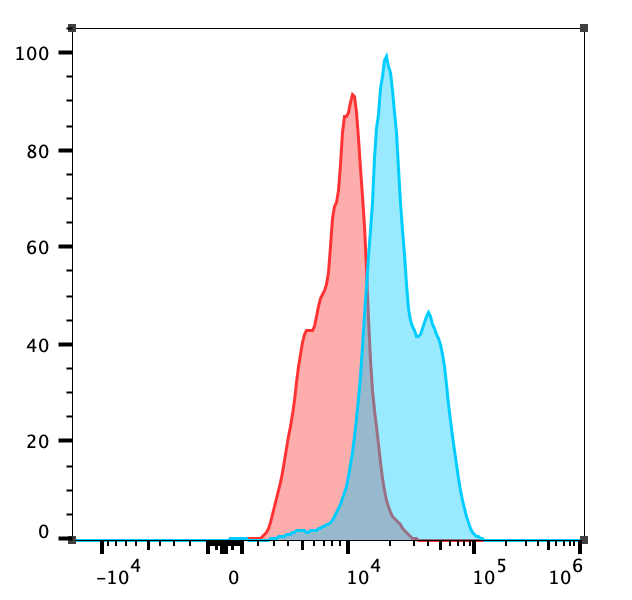 |
| --- | --- | --- | --- | --- | --- | --- | --- | --- | --- |
| **WM276**  **/VGI** | **WM179**  **/VGI** | **CBS8775**  **/VGI** | **C384/VGI** | **B4546/VGI** | **NIH312/VGI** | **EJB11**  **/VGI** | **MMCO8-897/VGI** | **CBS1508/VGI** | **CBC1873**  **/VGI** |

| **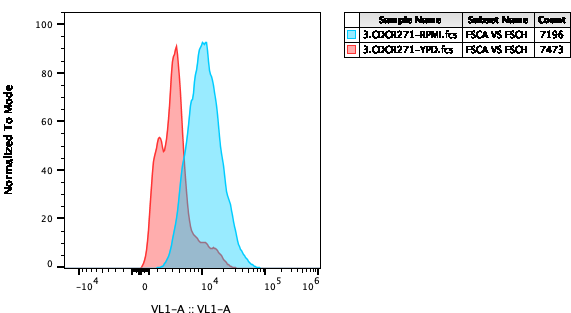** | **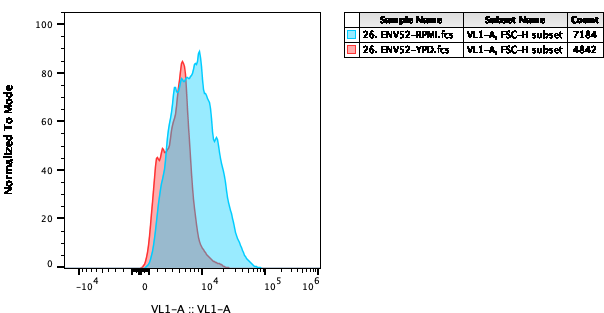** | **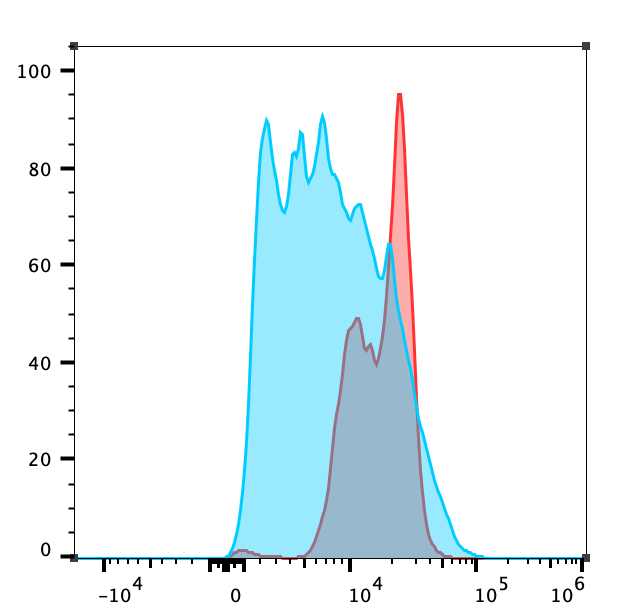** | **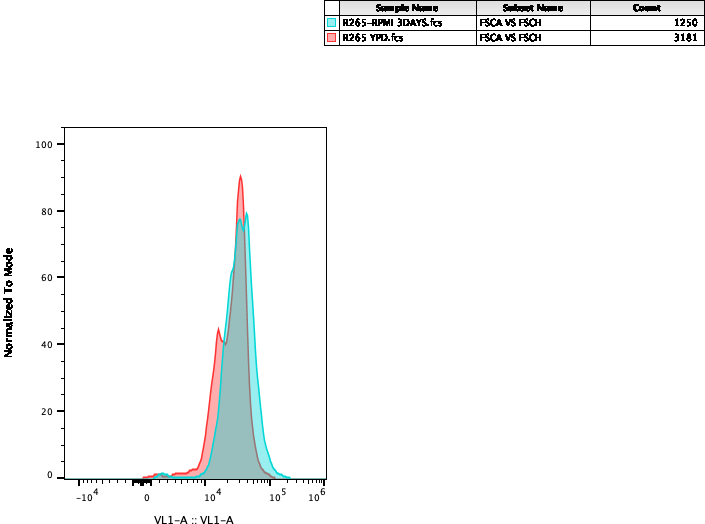** |
| --- | --- | --- | --- |
| **CDC271/VGIIa** | **ENV152/VGIIa** | **CDCF2866/VGIIa** | **R265/VGIIa** |

| **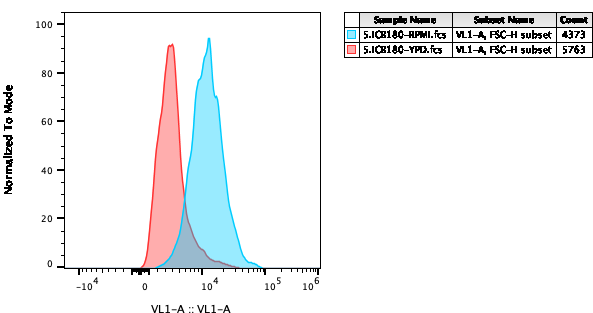** | **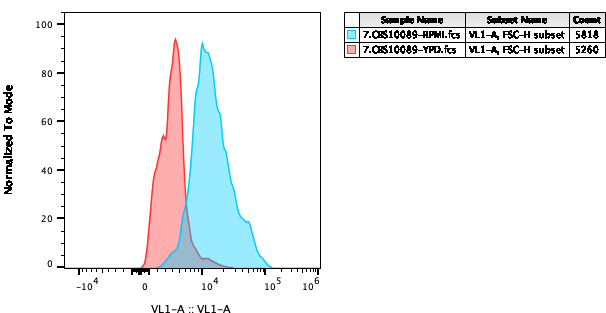** | **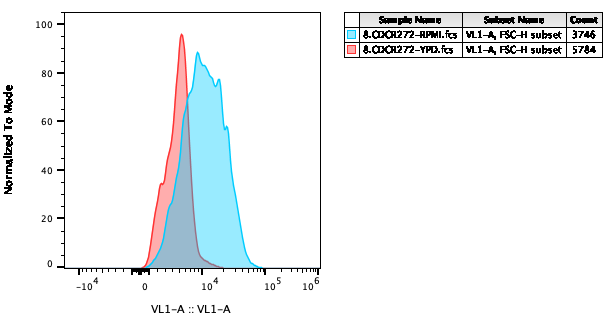** | **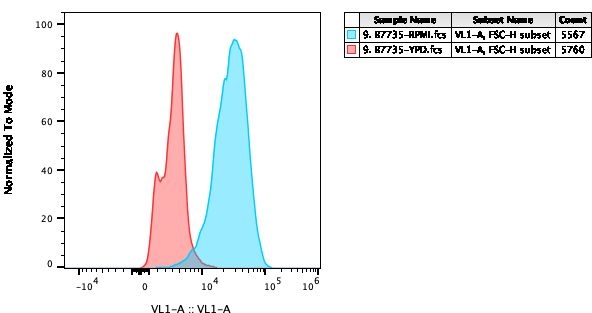** |
| --- | --- | --- | --- |
| **ICB180/VGII** | **CBS10089/VGII** | **CDCR272/VGIIb** | **B7735/VGIIb** |

| **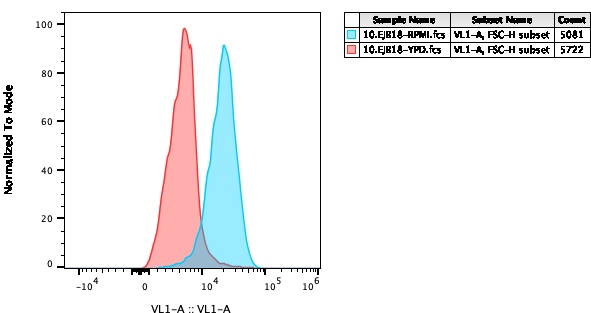** | **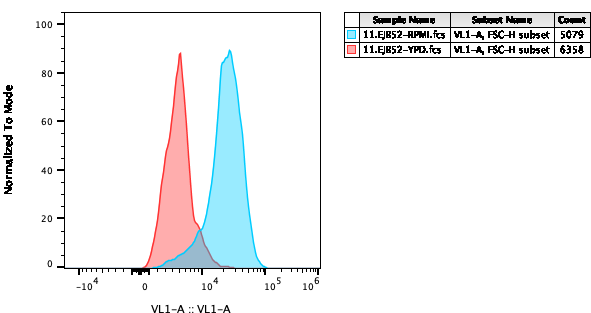** | **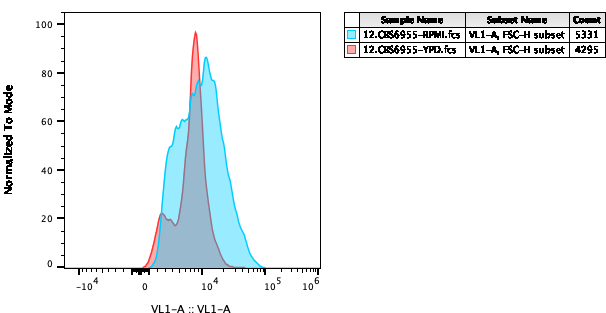** | **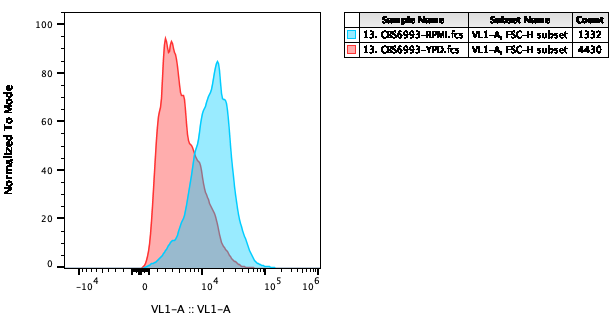** |
| --- | --- | --- | --- |
| **EJB18/VGIIc** | **EJB52/VGIIc** | **CBS6955/VGIII** | **CBS6993/VGIII** |

| 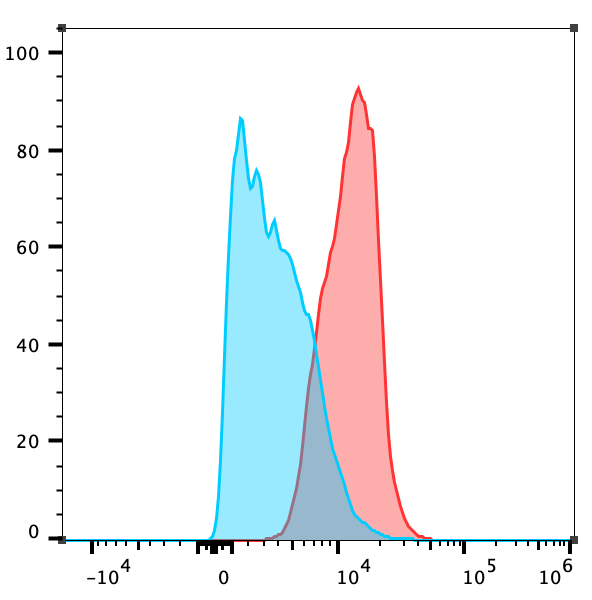 | 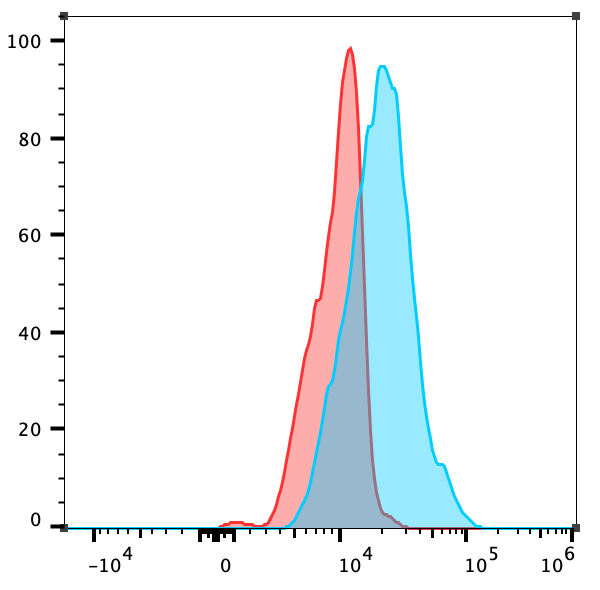 | 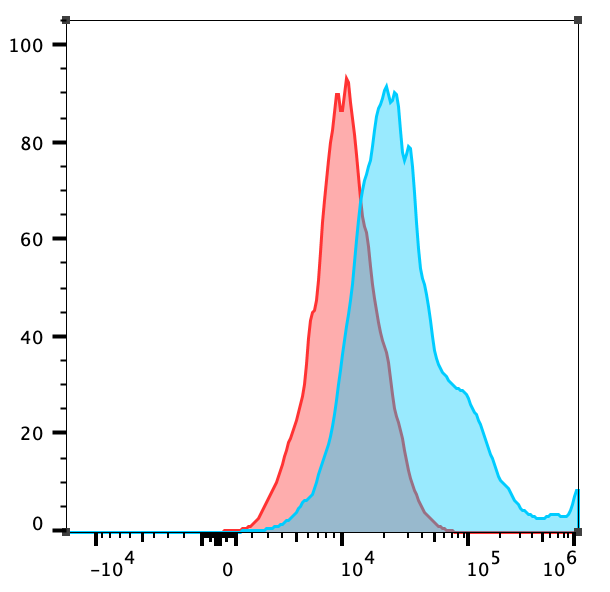 | 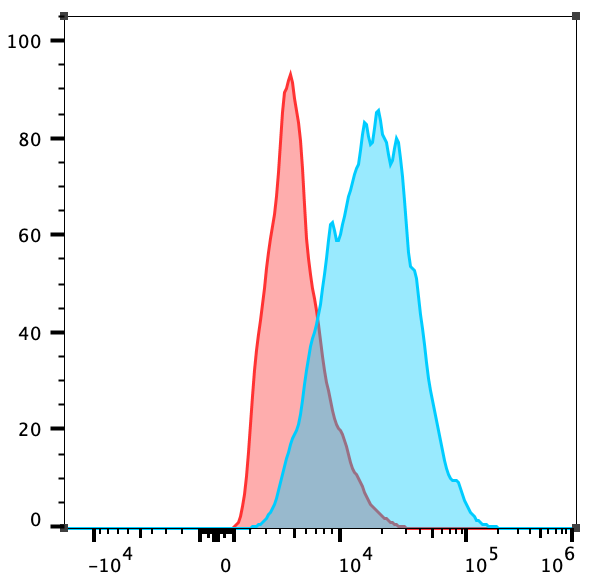 | 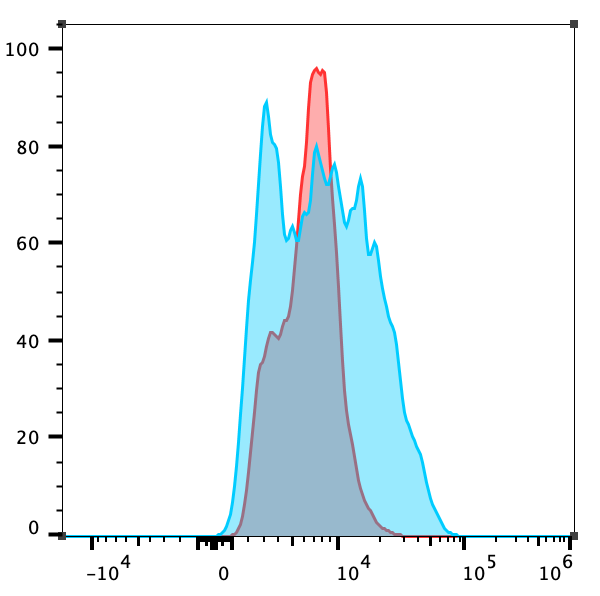 |
| --- | --- | --- | --- | --- |
| **CBS1622/VGIII** | **WM1243/VGIII** | **B13C/VGIII** | **CA2350/VGIII** | **CA1227/VGIII** |

| **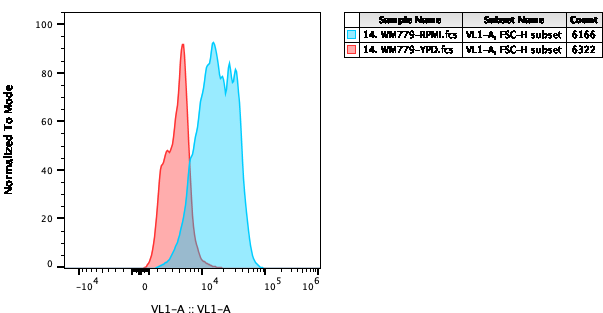** | **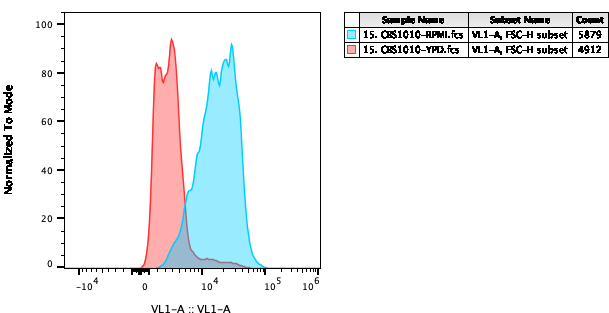** | **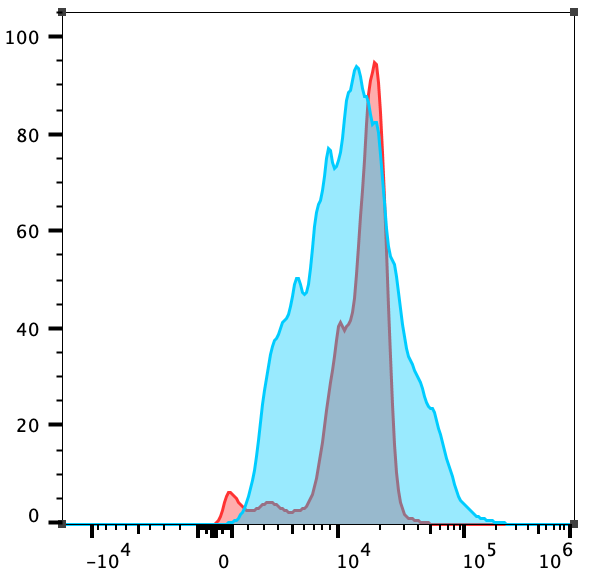** | **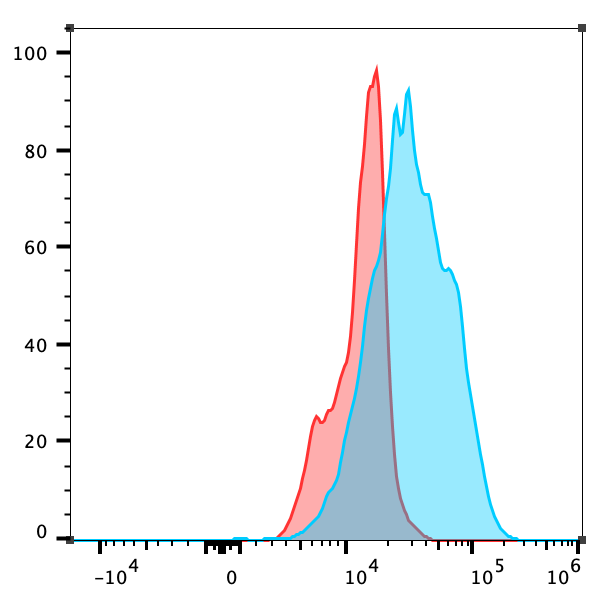** | **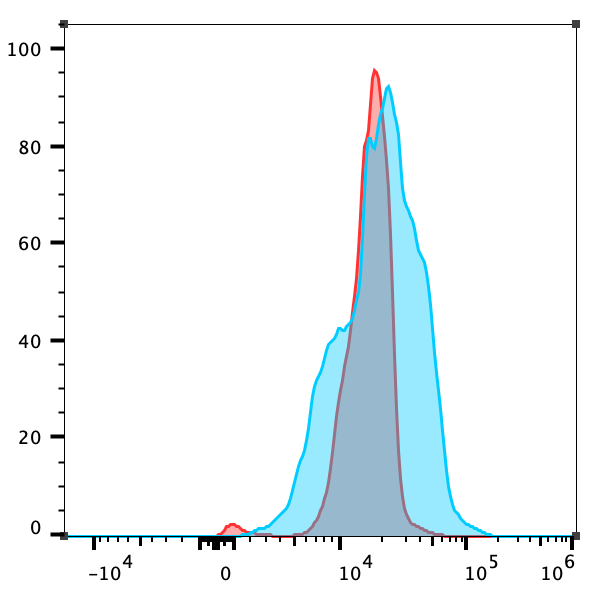** |
| --- | --- | --- | --- | --- |
| **WM779/VGIV** | **CBS1010/VGIV** | **B5742/VGIV** | **B5748/VGIV** | **CBS10101/VGIV** |

| **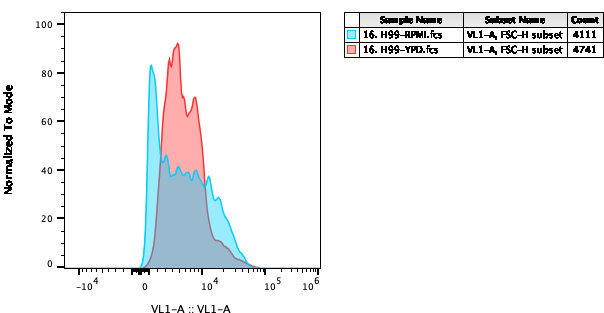** | **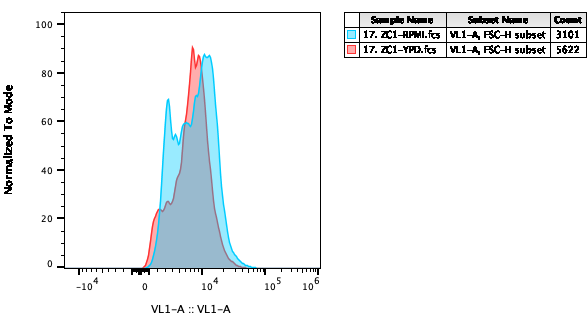** | **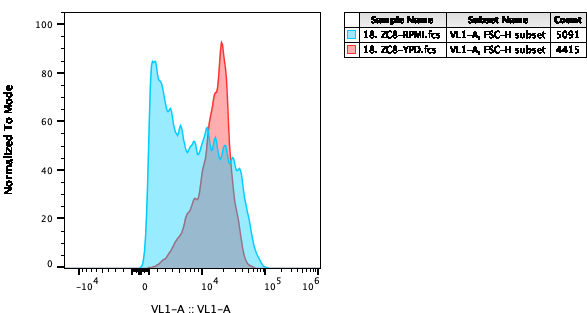** | **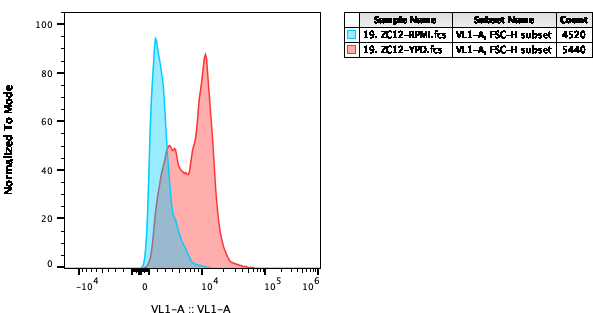** | **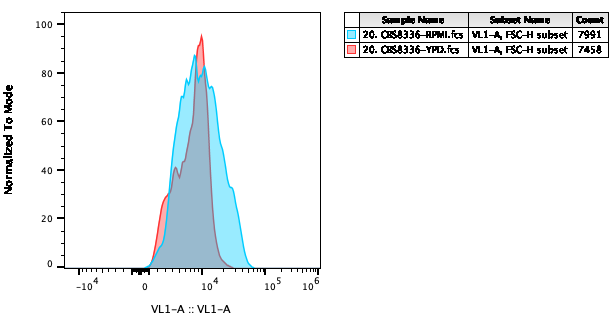** | **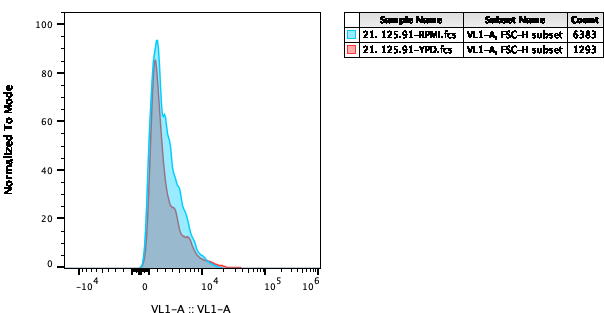** | **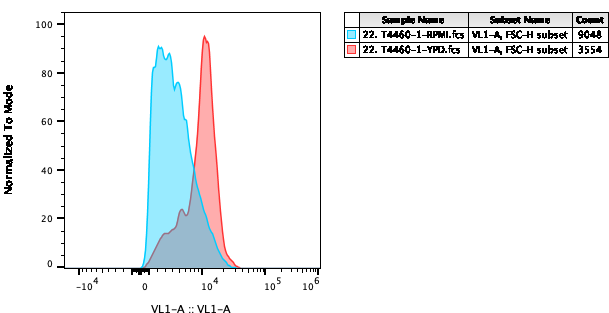** | **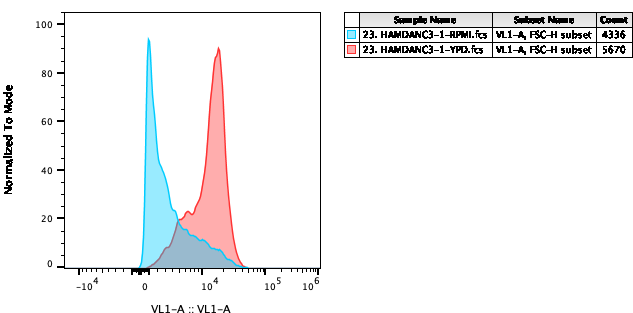** |
| --- | --- | --- | --- | --- | --- | --- | --- |
| **H99/VNI** | **ZC1/VNI** | **ZC8/VNI** | **ZC12/VNI** | **CBS8336/VNI** | **125.91/VNI** | **T4406-1/ VNII** | **HamdanC3'1/VNII** |

| **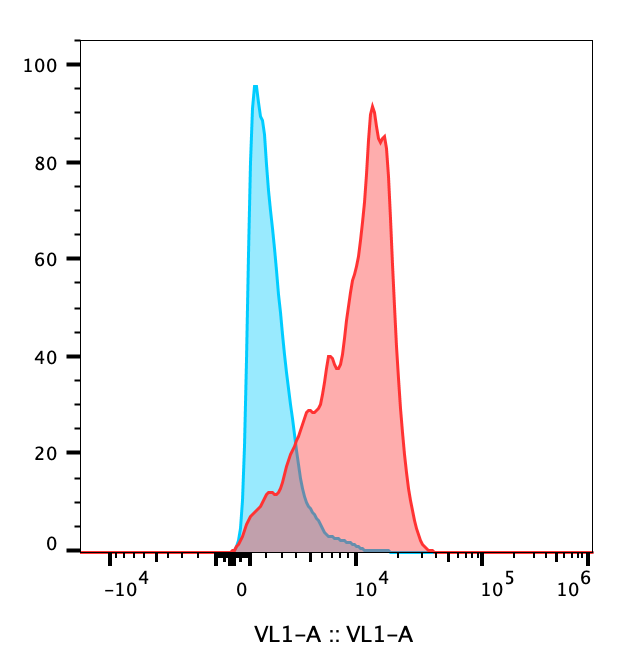** | **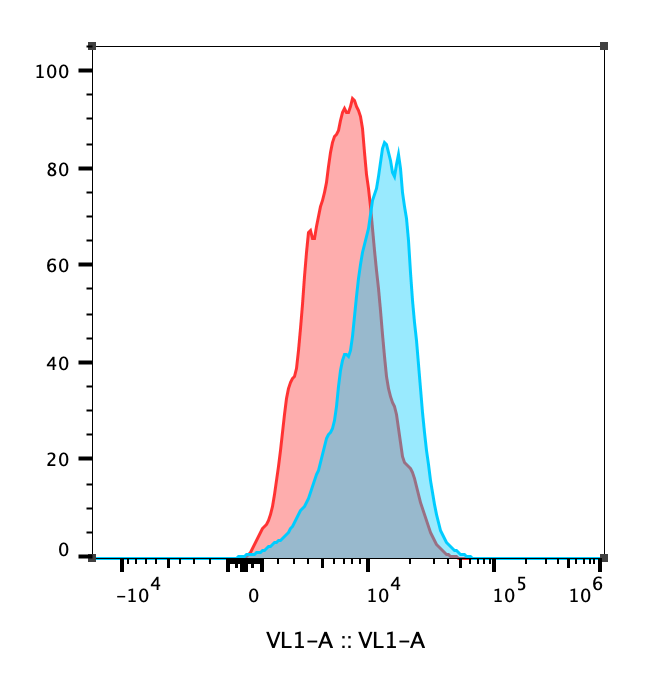** |
| --- | --- |
| **B3501/VNIV** | **CBS6995/VNIV** |
