# Supplementary material for: An in vitro method for inducing titan cells reveals novel features of yeast-to-titan switching in the human fungal pathogen Cryptococcus gattii
Source: PLoS Pathog. 2022 Aug 15;18(8):e1010321. doi: 10.1371/journal.ppat.1010321 (PMC9426920; doi:10.1371/journal.ppat.1010321)
Supplement: S6 Fig — A) DNA content of YPD grown (red) and titan-induced (blue) of ingroup crossing [VGII(R265) x VGII (LA584)] strains and their 13 progeny (Alg23-Alg35) after 3 days. All isolates were induced for titanisation according to our in vitro induction model (as mentioned in the Methods sections) after DNA content was confirmed by DAPI staining and flow cytometry analysis. B) DNA content of YPD grown (red) and titan-induced (blue) of ingroup crossing [VGII(R265) x VGIII (B4564) strains and their 18 progeny (P1-P18) after 3 days. All isolates were induced for titanisation according to our in vitro induction model (as mentioned in the Methods sections) after DNA content was confirmed by DAPI staining and flow cytometry analysis. (DOCX) [file ppat.1010321.s006.docx]

**Fig. S6 Ploidy of parent and progeny *C. gattii* strains before and after titan induction**

| **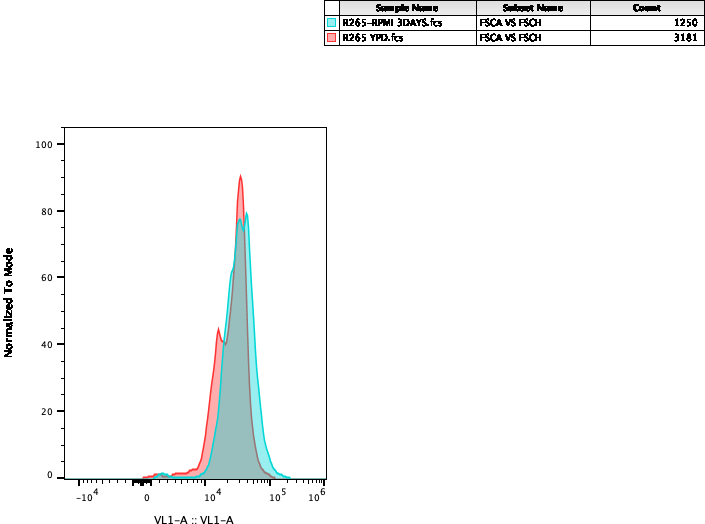** | 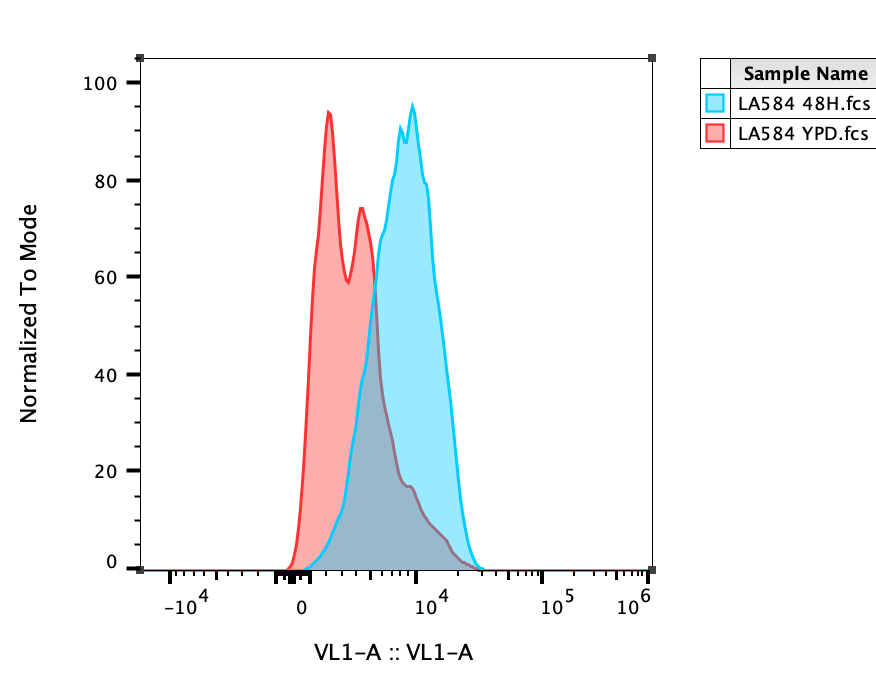 | **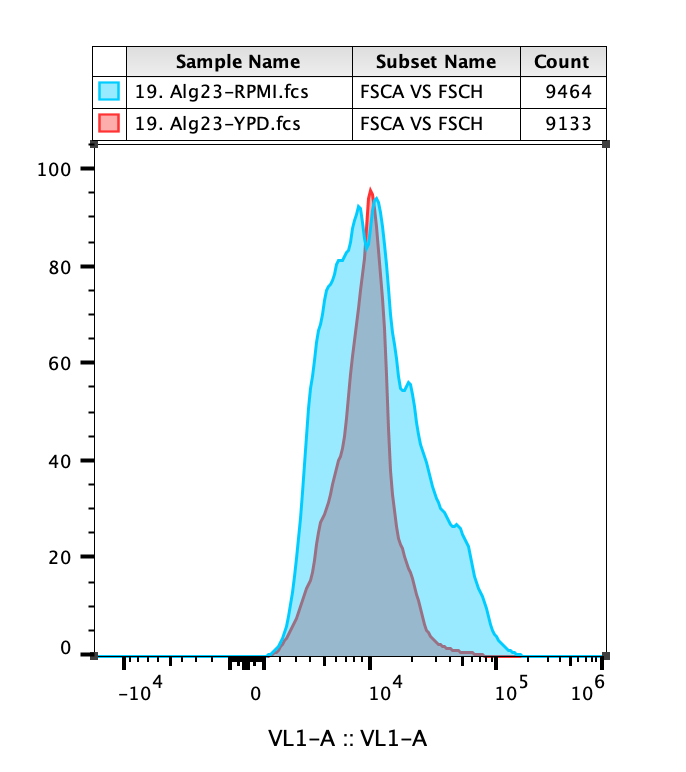** | **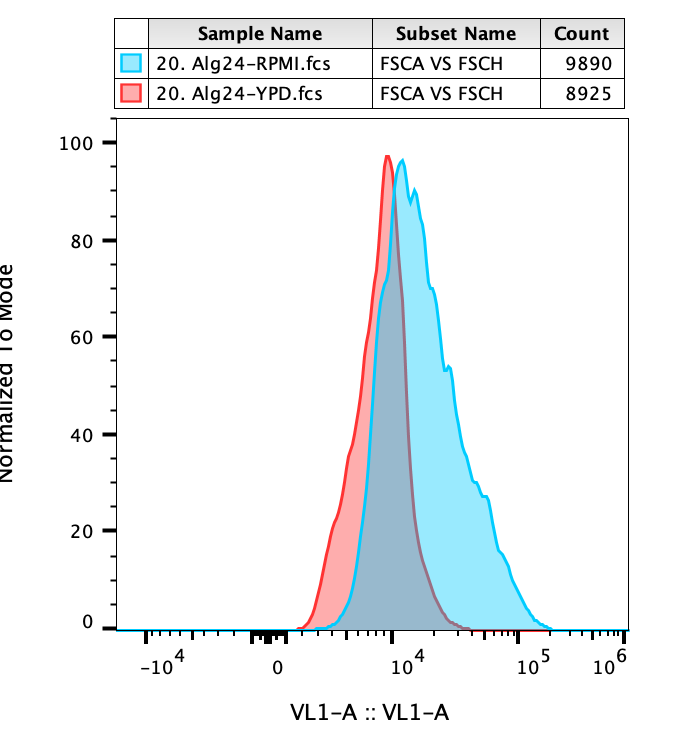** | **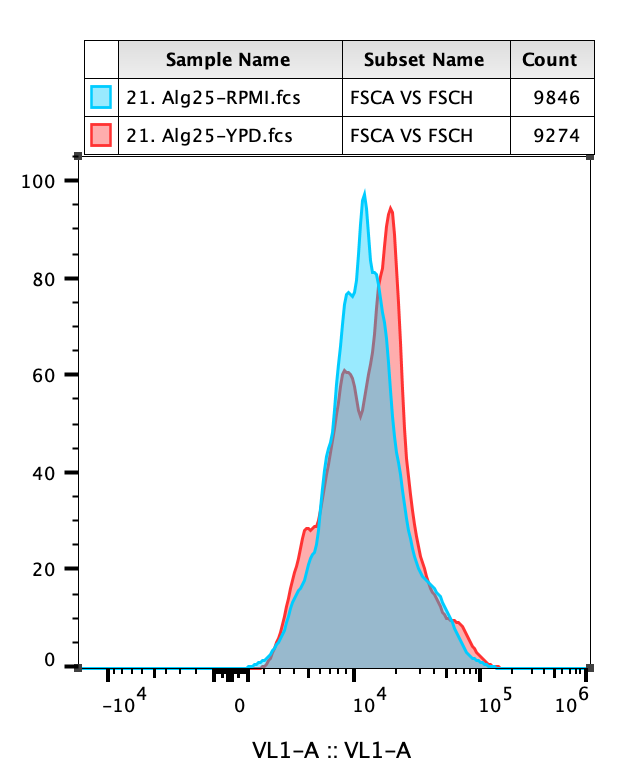** |
| --- | --- | --- | --- | --- |
| **VGII (R265)** | **VGII (LA584)** | **Alg23** | **Alg24** | **Alg25** |

| **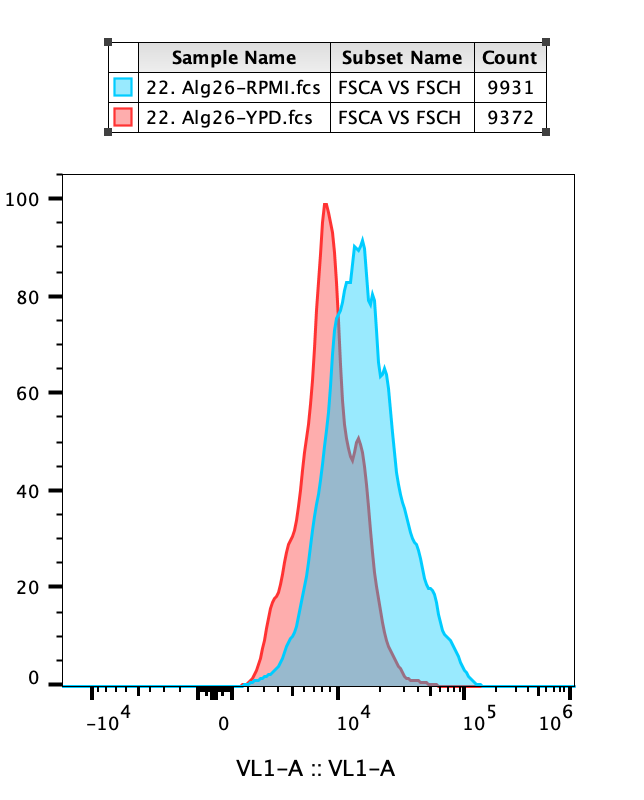** | **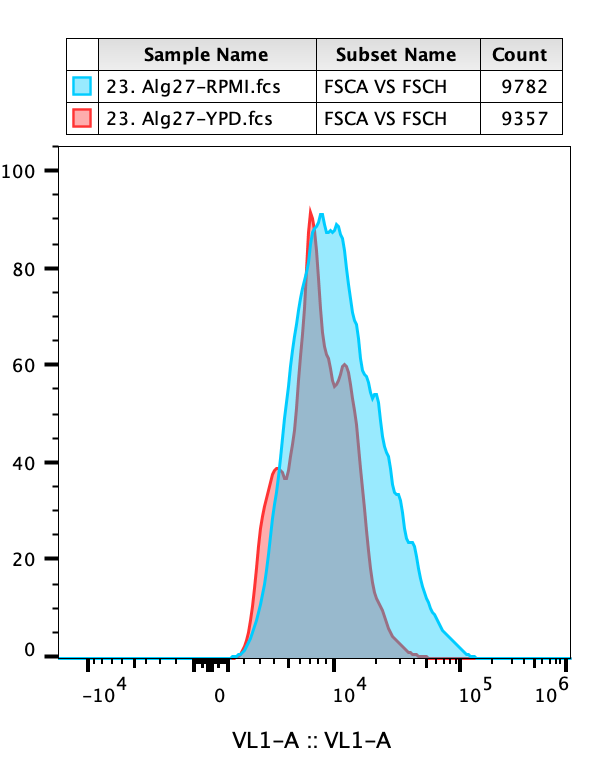** | **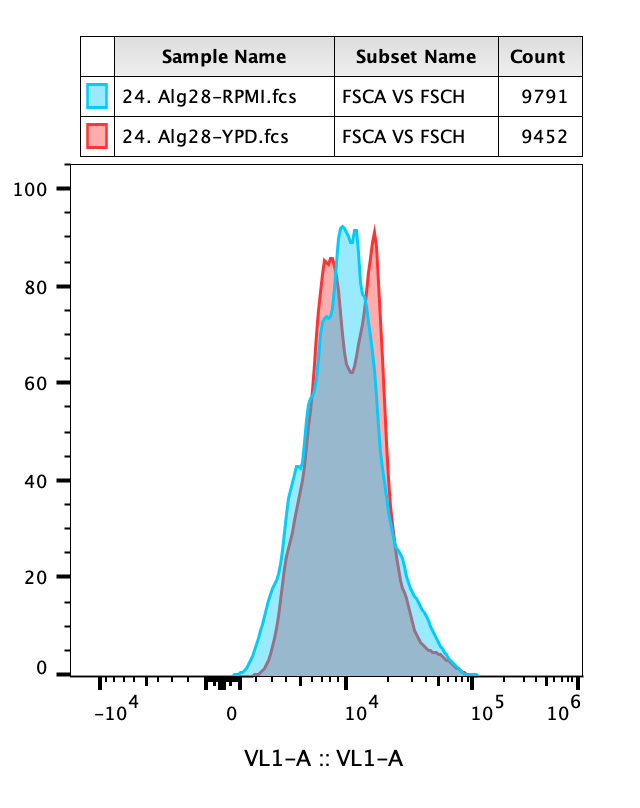** | **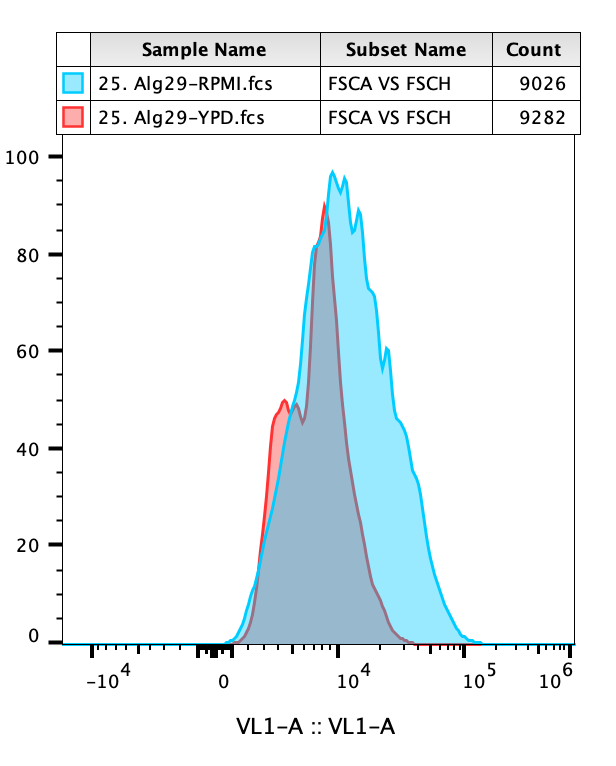** | **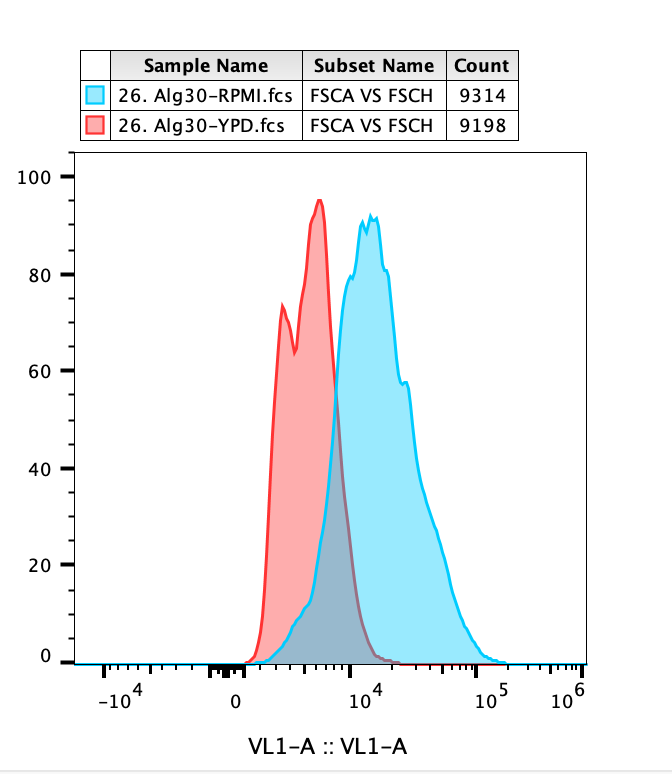** |
| --- | --- | --- | --- | --- |
| **Alg26** | **ALg27** | **ALg28** | **ALg29** | **Alg30** |

| **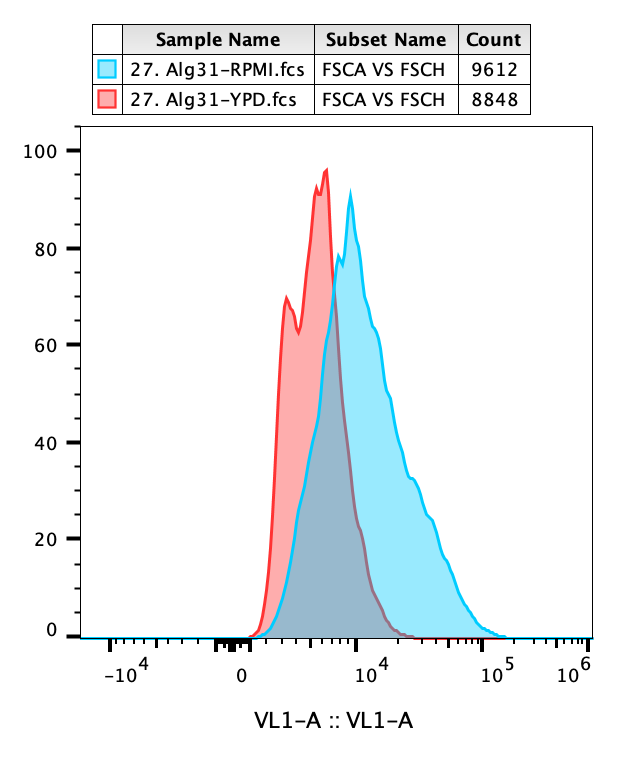** | **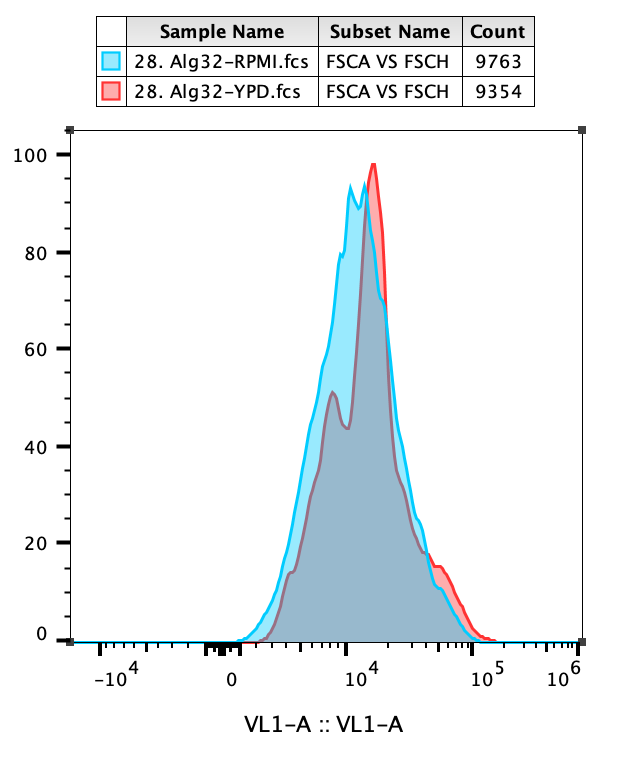** | **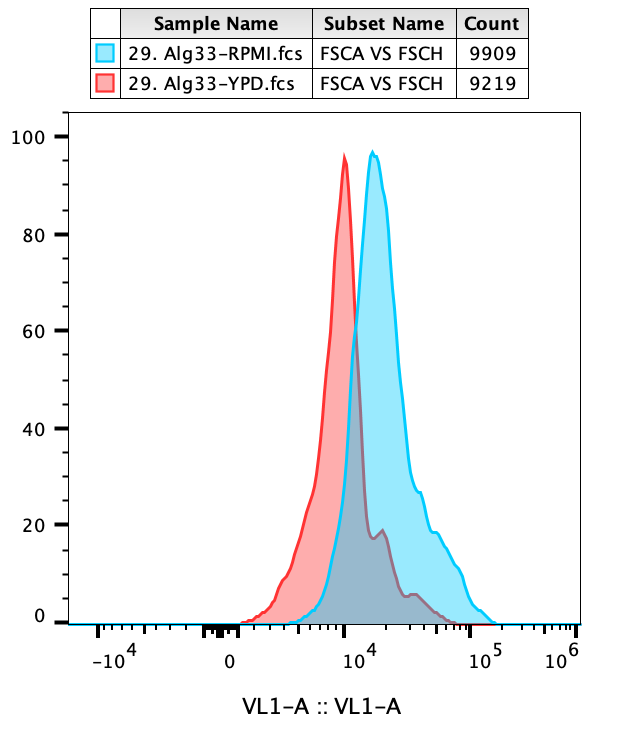** | **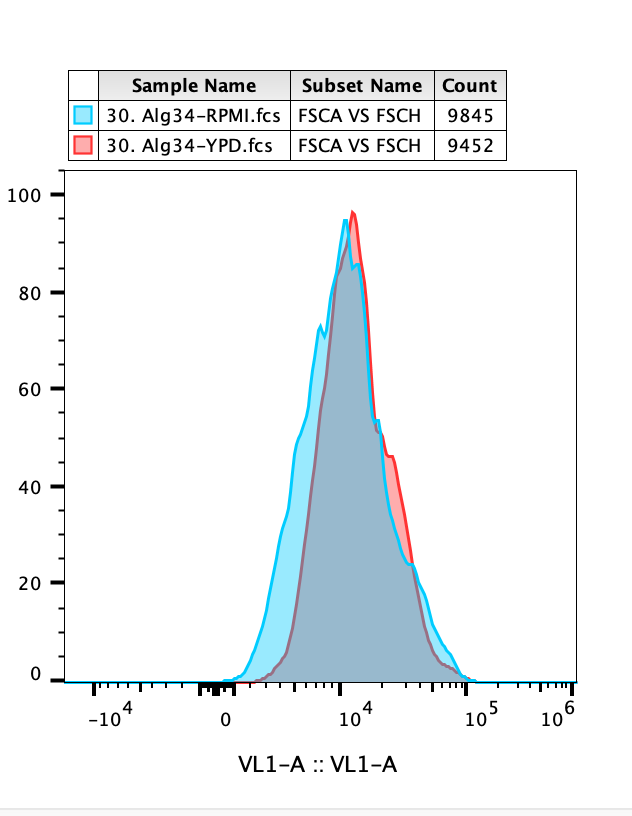** | **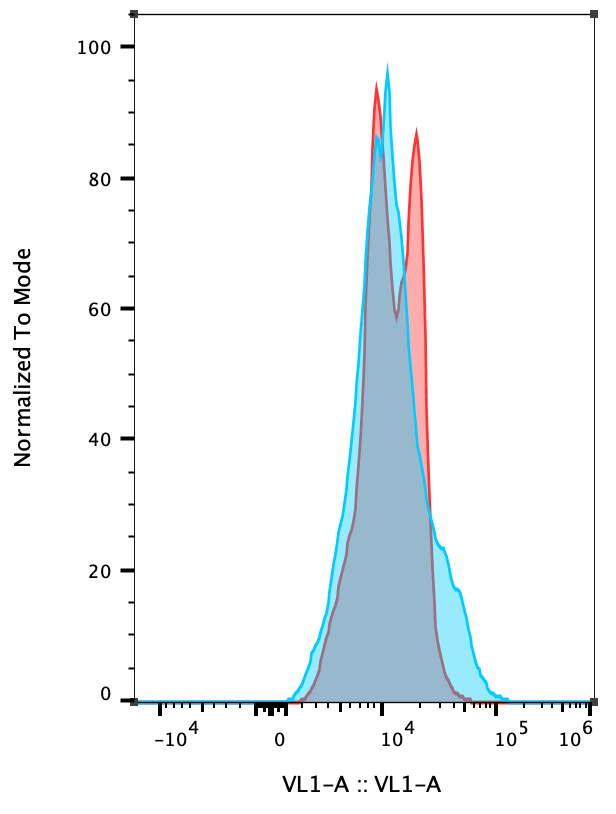** |
| --- | --- | --- | --- | --- |
| **Alg31** | **Alg32** | **Alg33** | **Alg34** | **Alg35** |

**A) DNA content of YPD grown (red) and titan-induced (blue) of ingroup crossing [VGII(R265) x VGII (LA584)] strains and their 13 progeny (Alg23-Alg35) after 3 days.** All isolates were induced for titanisation according to our *in vitro* induction model (as mentioned in the methods sections) after DNA content was confirmed by DAPI staining and flow cytometry analysis.

| **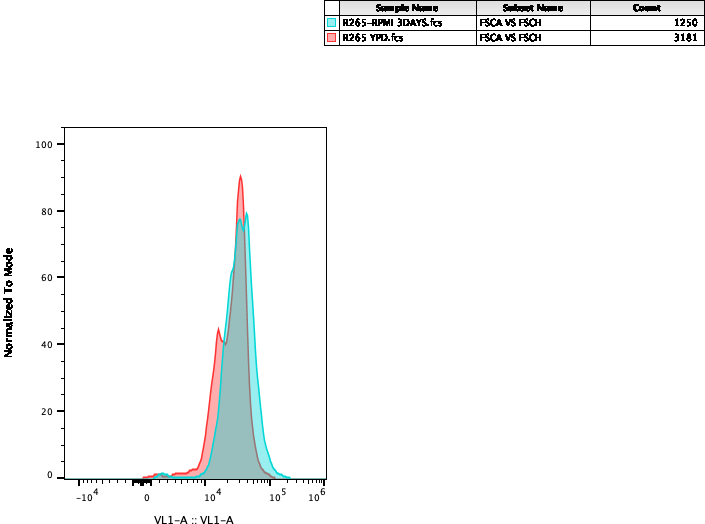** | **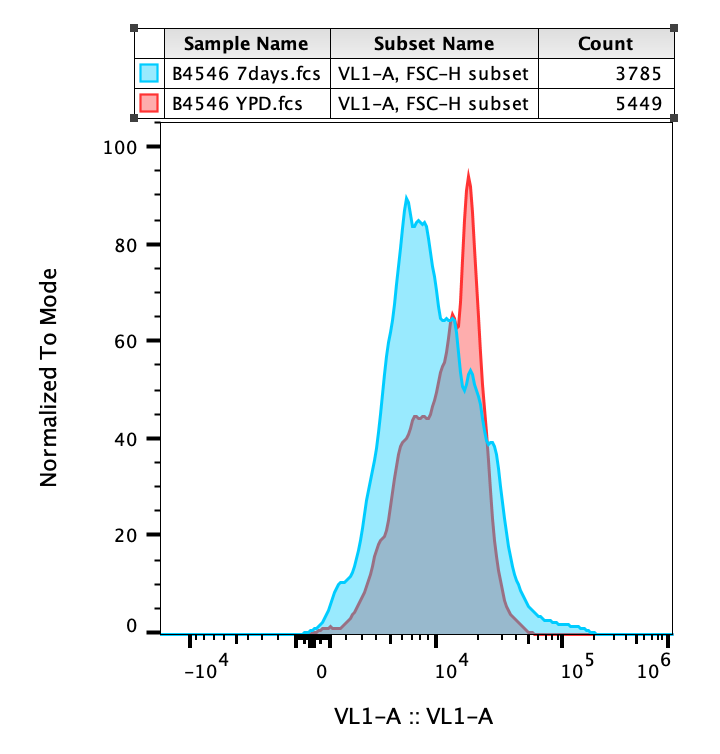** | **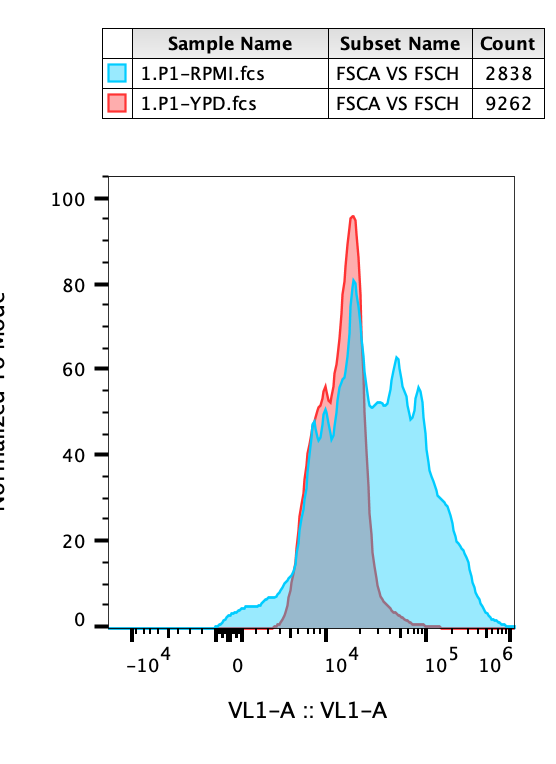** | **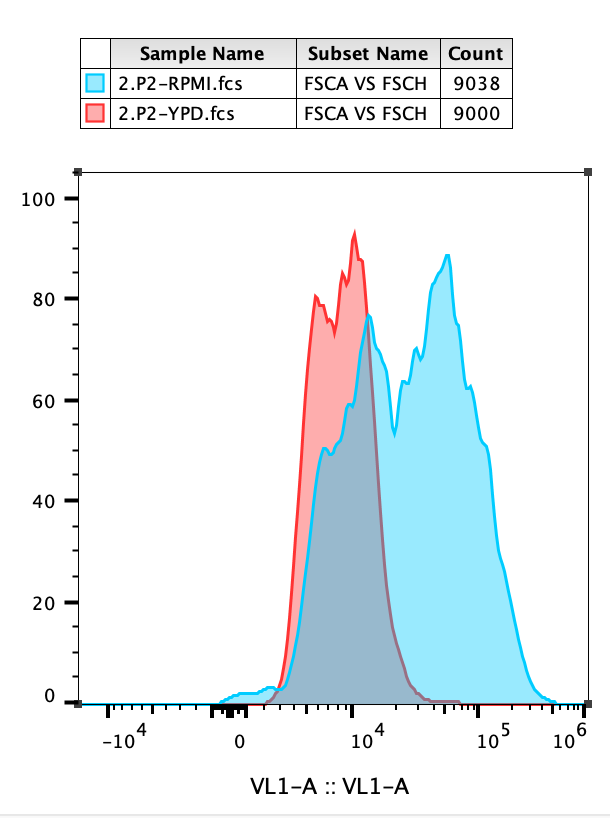** | 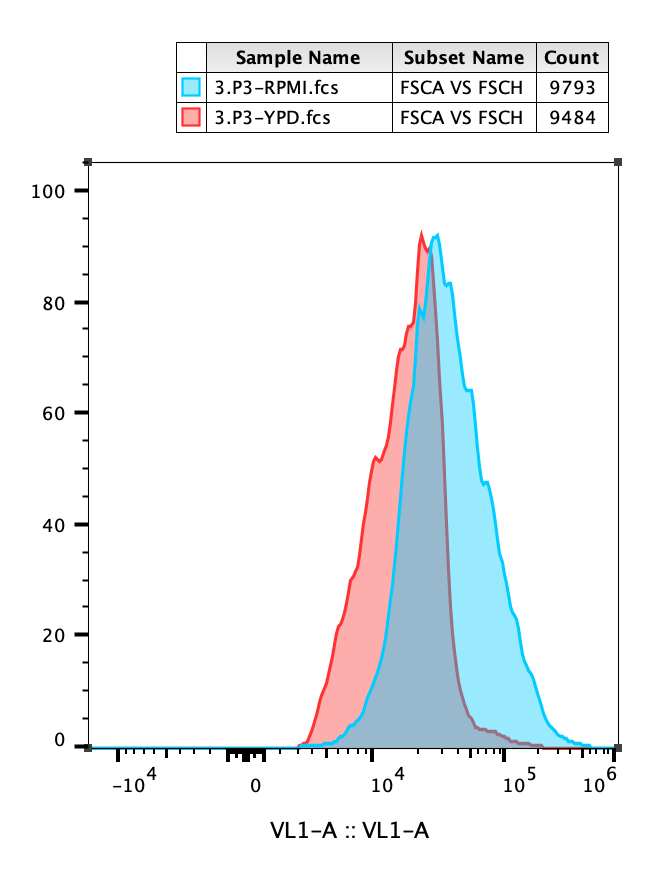 | 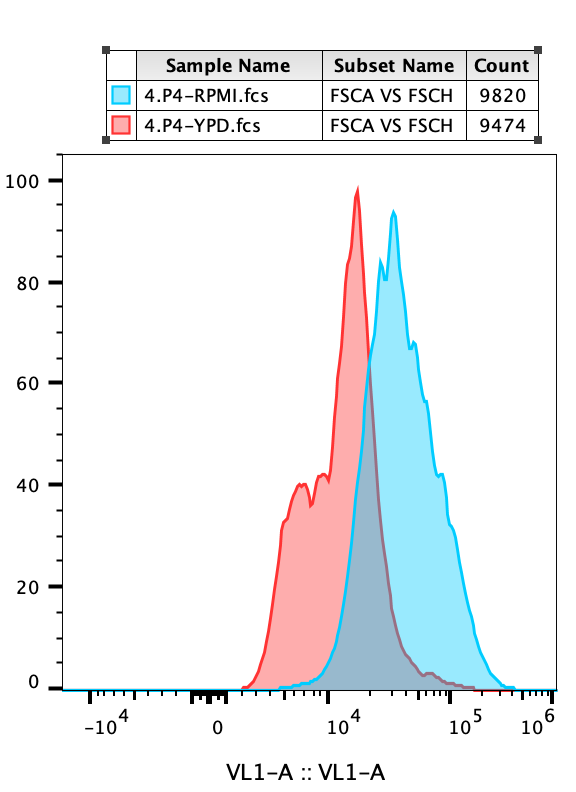 | **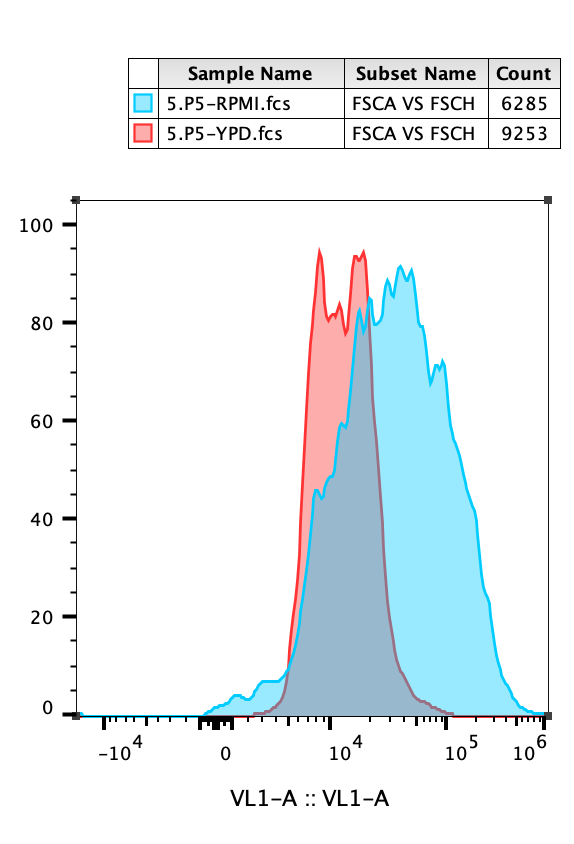** | **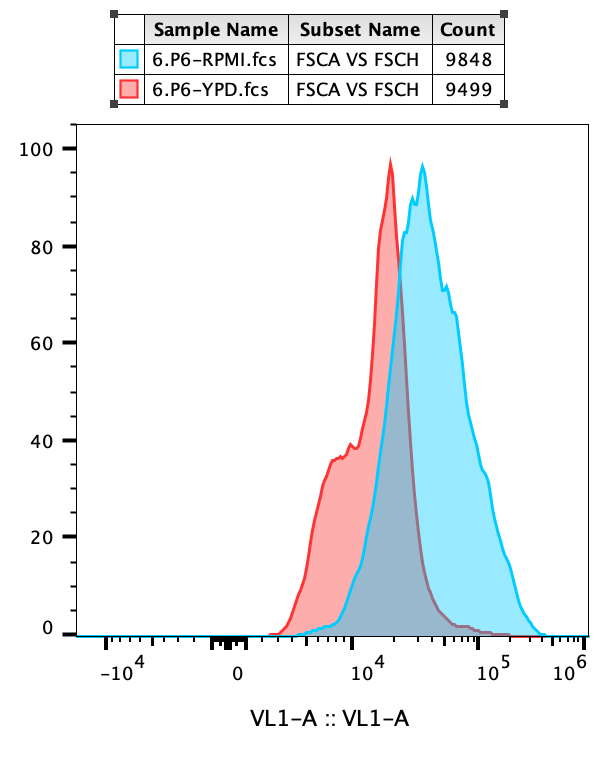** |
| --- | --- | --- | --- | --- | --- | --- | --- |
| **VGII (R265)** | **VGIII (B4546)** | **P1** | **P2** | **P3** | **P4** | **P5** | **P6** |
| **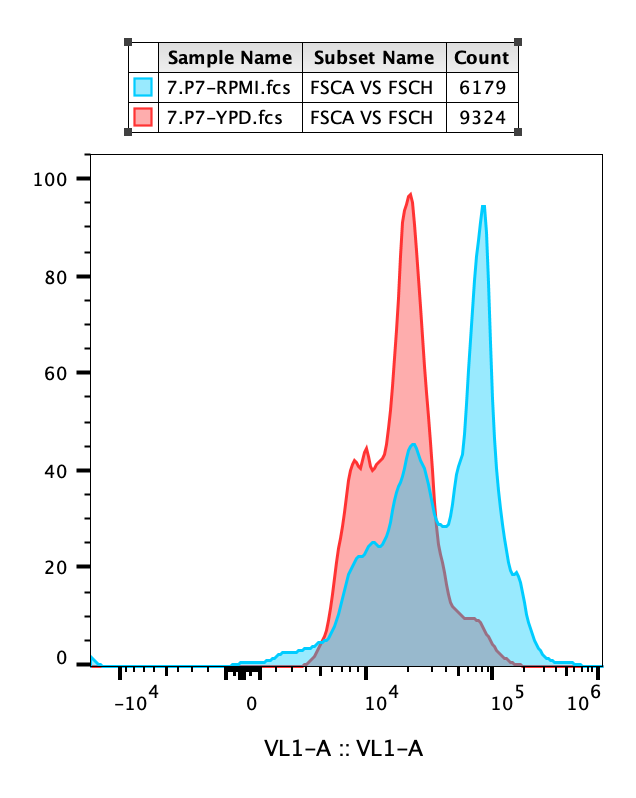** | **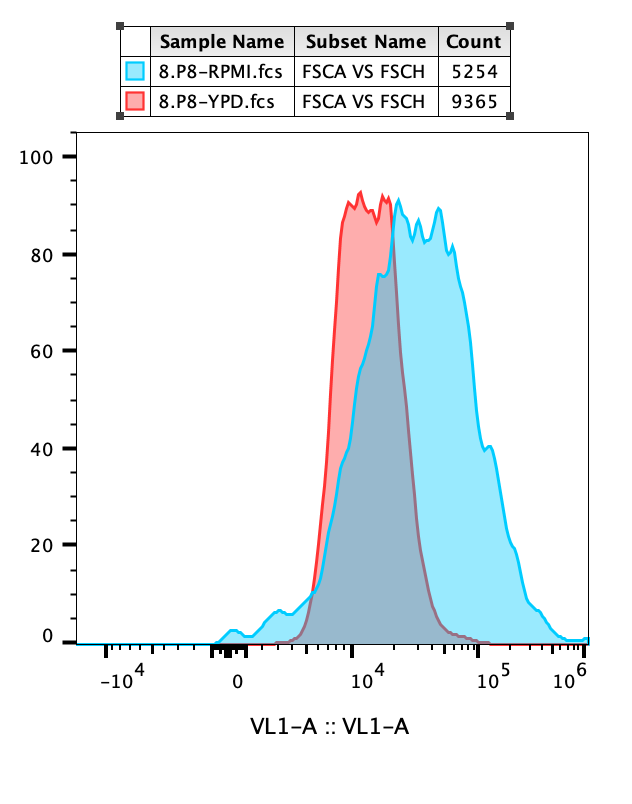** | **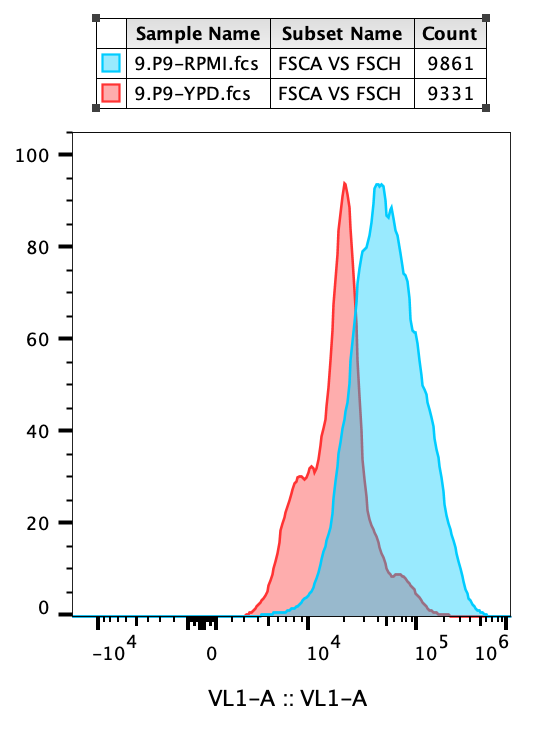** | 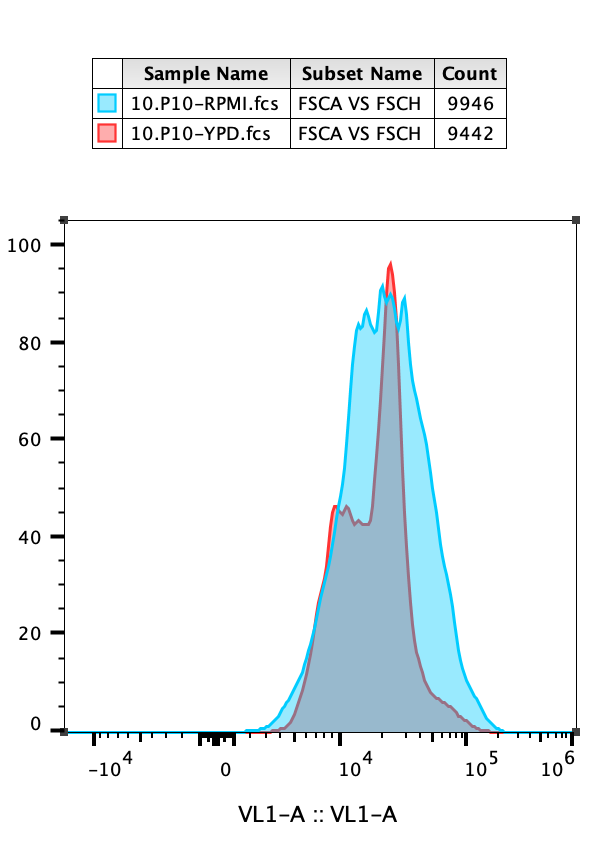 | 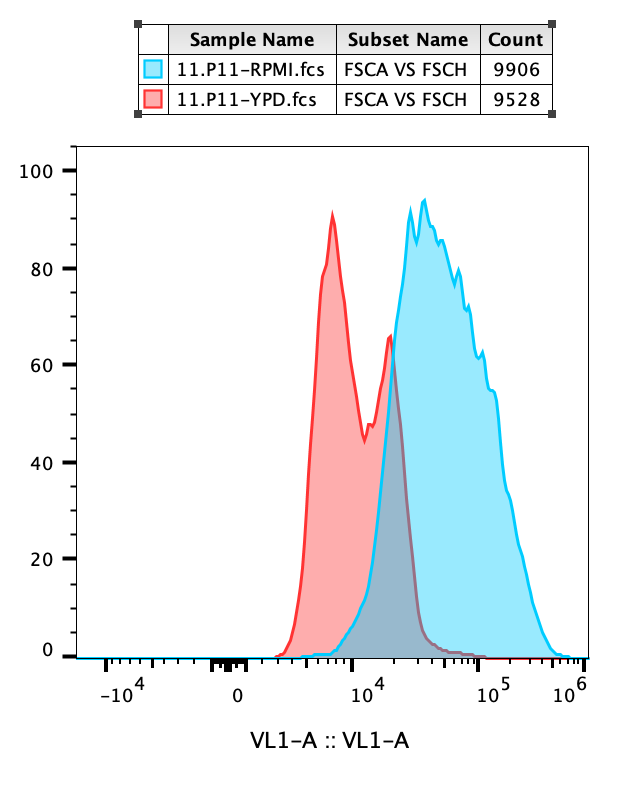 | **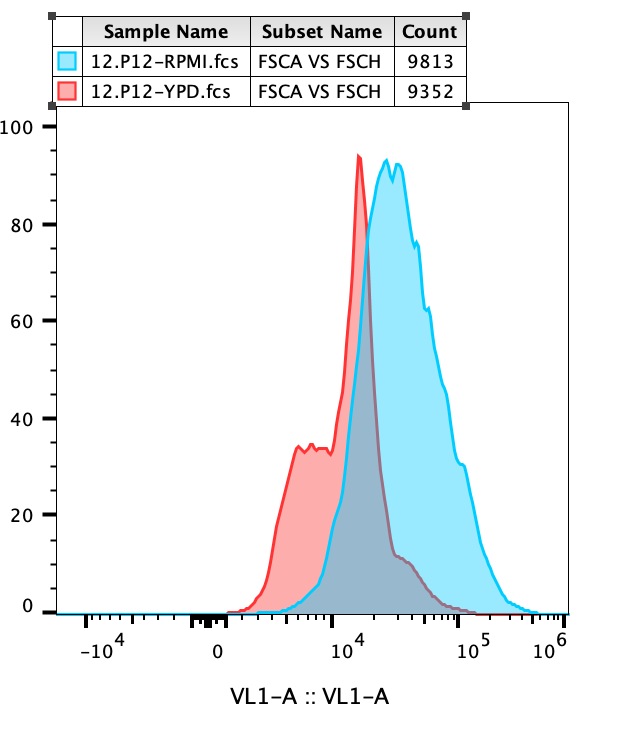** | **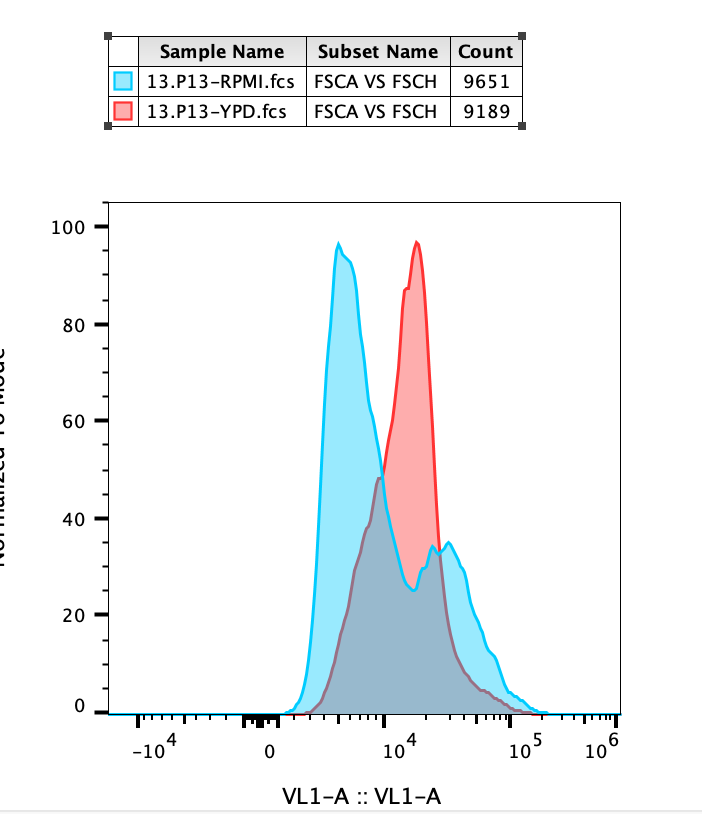** | **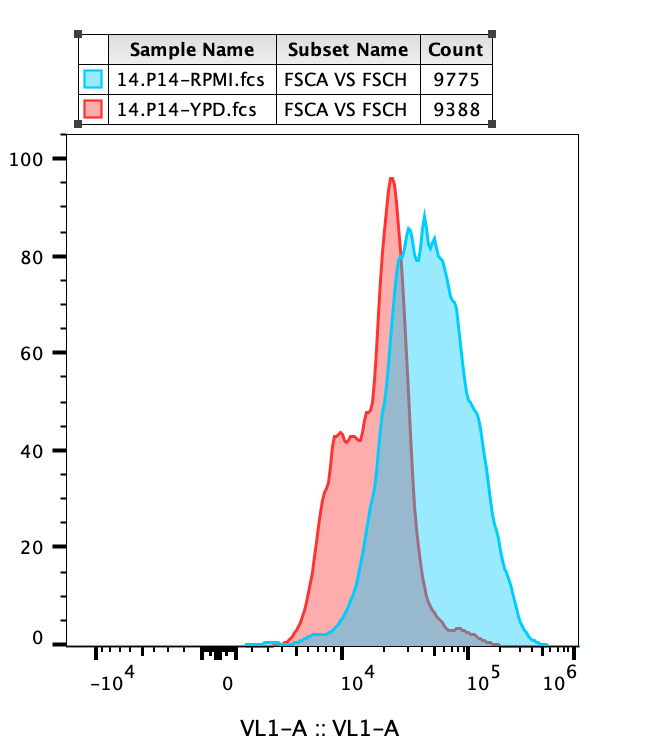** |
| **P7** | **P8** | **P9** | **P10** | **P11** | **P12** | **P13** | **P14** |

| **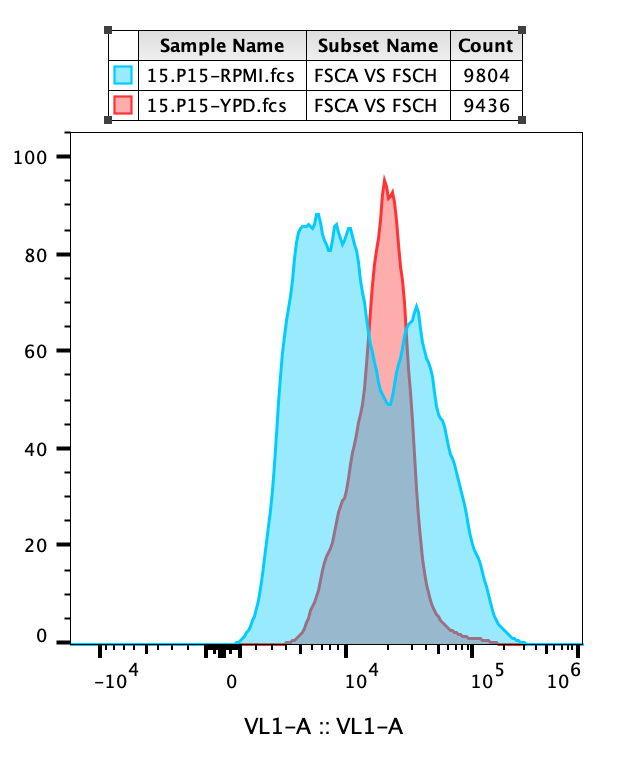** | **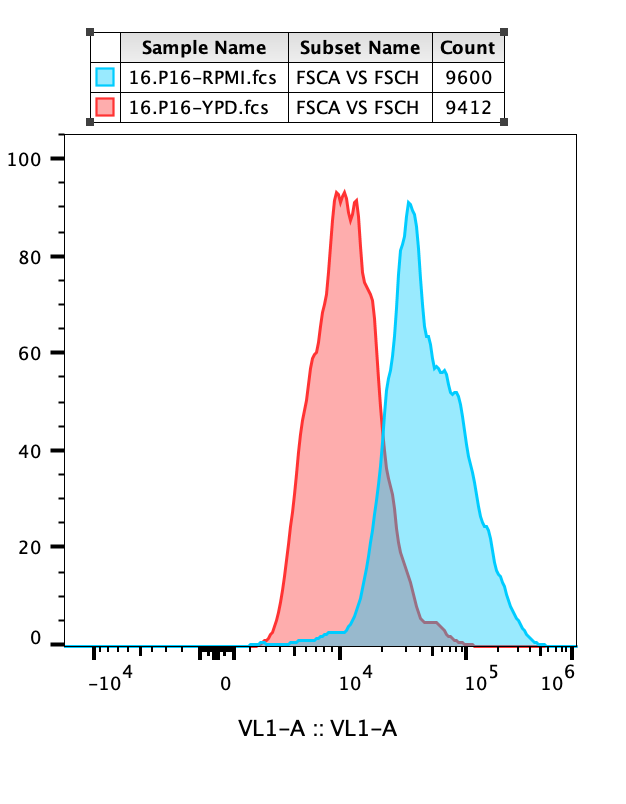** | **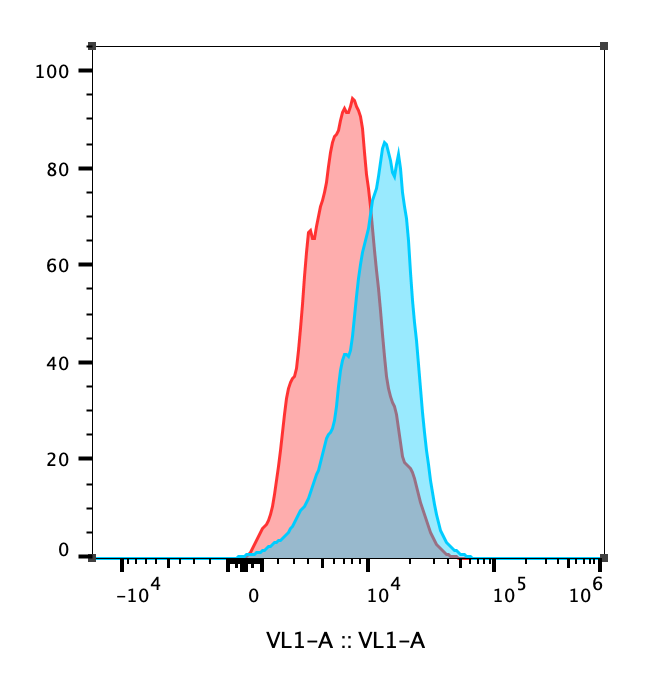** | **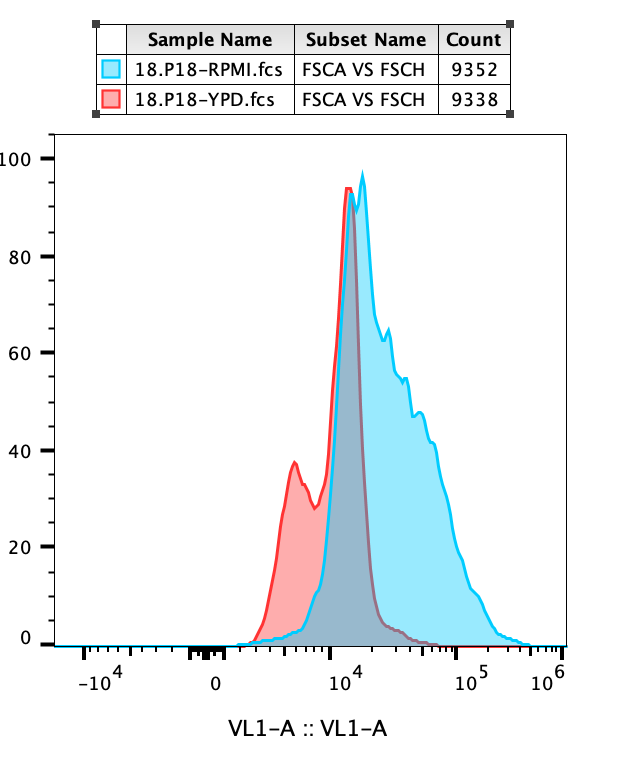** |
| --- | --- | --- | --- |
| **P15** | **P16** | **P17** | **P18** |

**B) DNA content of YPD grown (red) and titan-induced (blue) of ingroup crossing [VGII(R265) x VGIII (B4564) strains and their 18 progeny (P1-P18) after 3 days.** All isolates were induced for titanisation according to our *in vitro* induction model (as mentioned in the methods sections) after DNA content was confirmed by DAPI staining and flow cytometry analysis.
