# Supplementary material for: An in vitro method for inducing titan cells reveals novel features of yeast-to-titan switching in the human fungal pathogen Cryptococcus gattii
Source: PLoS Pathog. 2022 Aug 15;18(8):e1010321. doi: 10.1371/journal.ppat.1010321 (PMC9426920; doi:10.1371/journal.ppat.1010321)
Supplement: S7 Fig — Microscopy images of YPD grown (A) and 72 hr titan-induced C. neoformans cells (B). Scale bar = 5μm. (DOCX) [file ppat.1010321.s007.docx]

Fig. S7. **Morphology of *C. neoformans* cells before and after titan-induction via our *in vitro* model**. Microscopy images of YPD grown (A) and 72 hr titan-induced *C. neoformans* cells *(*B). Scale bar=5µm.


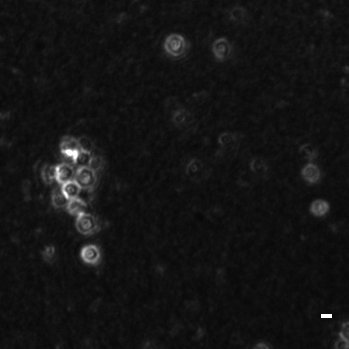

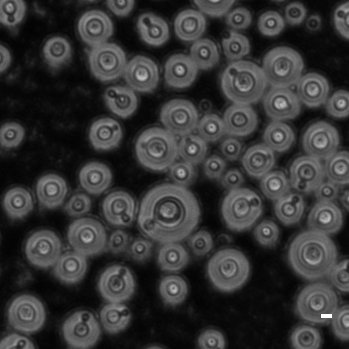


**A B**
