# Supplementary material for: An in vitro method for inducing titan cells reveals novel features of yeast-to-titan switching in the human fungal pathogen Cryptococcus gattii
Source: PLoS Pathog. 2022 Aug 15;18(8):e1010321. doi: 10.1371/journal.ppat.1010321 (PMC9426920; doi:10.1371/journal.ppat.1010321)
Supplement: S1 Table — (DOCX) [file ppat.1010321.s008.docx]

| **Induction time points** | **Phenotype/Cell cycle phase** | **Cyclin gene in C. neoformans (*S. cerevisiae*)** | **Orthologous gene in R265 (function)** | **Primers** |
| --- | --- | --- | --- | --- |
| 24 hr | Bud formation/**G1** & **M** | CNAG_02196 (CDC11) | CNBG_5339 (Septin) | GTCATTCCCGTCATTGGCAA  ATCTTCCTCGGCGTCATAGG |
| 3 days | Mitosis exist/ **G1/S** | CNAG_06092 (CLN1) | CNBG_4803 (Cyclin 1) | AGCTCCCAACGGTAGTATCG  ATGTGGGAAGTGATGTCGGT |
| 5 days | DNA REPLICATION/**S** | CNAG_03962 (MCM6) | CNBG_5506 (minichromosome maintenance protein 6) | CGAGCAAACTGTCAACACGA  TCAGTTGTGGGTCCTCATCC |
| 7 days | G2 arrest | CNAG_05406 | CNBG_4446 (mitotic check point protein bub2) | ACCAGCCACCCATCTACTTC  CTGCGTATCCTTTTGAGGCC |
| Gene control  GAPDH |  |  | CNBG_1866glyceraldehyde-3-phosphate dehydrogenase | GAAGGTCGTCATTTCCGCTC  GCTTGTAGGCATCGAGGTTG |

Table S1. List of cell cycle phenotypes of titan induced and associated genes
