# Supplementary material for: An in vitro method for inducing titan cells reveals novel features of yeast-to-titan switching in the human fungal pathogen Cryptococcus gattii
Source: PLoS Pathog. 2022 Aug 15;18(8):e1010321. doi: 10.1371/journal.ppat.1010321 (PMC9426920; doi:10.1371/journal.ppat.1010321)
Supplement: Attachment — Submitted filename: Point by Point Response.docx [file ppat.1010321.s010.docx]

**Point by Point Response to the Reviewers**

**Editor’s Comments**

The editor highlighted two aspects, in particular, which had been raised by the reviewers:

**1. Specifically, the production of titan cells by *C. gattii* has already been demonstrated and it was felt that the authors did not provide sufficient comparison to *C. neoformans* titan cell formation to show that the differences in induction of titan cells they observed with *C. gattii* would not also occur with *C. neoformans*. As one example, without assessing the role of p-aminobenzoic acid in *C. neoformans* titan cell production it is difficult to determine the novelty of this finding to *C. gatti*i.**

*We now include data on the response of several C. neoformans strains to our titanising protocol (Table 1) and specifically test the response of C. neoformans H99 to p-aminobenzoic acid (Supplmentary Figure 1E). It is interesting that the titanising capacity of H99 in this in vitro method is very low, and yet p-ABA is still able to moderately enhance this response; something that we now include in the revised discussion.*

**2. In addition, the data relating to the metabolic state of the cells, and whether the cells analyzed were in fact dead, raised additional questions about the novelty of these findings.**

*Our revised manuscript includes extensive new data demonstrating that titan cells are alive and metabolically active. Specifically, we show data using the metabolically-responsive dye FUN-1 in new Figure 5E, we demonstrate the production of daughter cells from titan ‘mother cells’ in Figure 5A-D, and we show timelapse imaging of budding from titan cells in Video S1.*

**MAJOR POINTS**

**Reviewer 1**

**1. Incubation in YPD at 37°C and 5% CO2 (24H and 7 days) as a control condition of titan cell formation would be a nice addition to the author’s experiments.**

*These data are now included in revised Figures 1 and 4.*

**2. Line 87-88: could you add details on the use of serum in the first induction medium as in cell culture medium, fetal calf serum is often added to help mammalian cells to grow.**

*Now added.*

**3. Line 115: All in vitro protocols evidenced a large vacuole occupying the cytoplasmic space.**

*Correct. This large vacuole is characteristic of bona fide titan cells and we now make this clear in the revised text.*

**4. Line 129-130: Fig 1E: the 3 large cells are looking as dead cells. Indeed, the nice phenotype inclufing the large vacuole is not visible on this picture. These cells have been characterized in Dromer’s lab pmid: 25827423 and look similar.**

*We now include multiple lines of evidence that these titan cells are alive (Figure 5 and Video S1) and have also updated this figure to avoid confusion.*

**5. Please use merge picture (DIC+DAPI) for DAPI stainings. This will better show that the DAPI staining does not stain a nucleus but rather the remaining retracted cytoplasm of this dead cell. This point is important, because to describe a population with 16C, the authors should be sure that the staining is done on a population harboring living cells in a good shape.**

*We now include new images, including a merged image, in Figure 5, as requested. We also include multiple lines of evidence (metabolic activity and timelapse imaging of proliferation) to demonstrate that titan cells are alive – see response above.*

**6. More pictures of 7 days incubated C. gattii using DIC should be provided. Fig 1A are only showing 24h-incubated cells.**

*Now added (to Figures 2, 3 and 4).*

**7. Fig 4E: The authors should be sure that no “contamination with titan cells” occurs in their setting to avoid detecting polyploid Titan cells within the population of daughter cells. Please analyze and provide histograms of all population (in SSC and FSC channels) to assess the absence of big cells from the daughter cells purification.**

*We have now undertaken additional flow cytometry, now included as new Figure 5, along with histograms and the inclusion of our full gating strategy as supplementary material.*

**8. Line 238-241. It is not clear in the text which conditions have been used for RNASeq analysis. Between 24h and 7days or at 24h and 7d?**

*This analysis was qRT-PCR, rather than RNA-Seq, and was conducted at 24hrs, 3, 5 and 7 days post-induction. This is now made clear in Figure 6.*

**9. Figure 6 (impact cell density and temperature and CO2) should appear earlier in the results. As these conditions should be fixed rapidly to standardize experiments and obtained the optimal Titan cell proportion.**

*We now include cell density data in Figure 3 and temperature/CO2 data in Figure 4, along with additional supplementary data on this point.*

**10. Line 323: should be 6F instead of 6B**

*Corrected*

**11. Fig 6G and 6H should be separated from the rest of Fig 6.**

*Now done.*

**12. Fig 6H. why using 3 h rather than 7 days as the best time point to obtain a large number of TC.**

*3 days (not hours) was used for practical reasons. Since we were comparing treatments, we did not need to chose ‘maximal’ titan cell formation, but simply a time point at which we could measure relative efficiency of producing titans.*

**13. The authors should investigate the pH of the different media tested. pH has an impact on Titan cell formation.**

*We have measured the pH of the different media used and include these data in Supplementary Figure 1.*

**14. Fig 6H that authors should investigate why pABA acts as an inducer of TC.**

*We now include additional data on the impact of pABA on titan induction, including demonstrating that pABA moderately enhances the (very low) titanisation capability of C. neoformans in this assay (Supplementary Figure 1). We respectfully suggest that a detailed investigation of the molecular pathway that leads pABA to trigger titanisation is beyond the scope of this current work, although clearly is of major interest for future research.*

**15. As the authors tend to show that VGII produce more TC than the other lineages, they should include more strains to validate this observation on more than 9 cells. They also should include at least the same number of strains from the other lineages (only 2 strains for VGI or VGIII, VGIV).**

*We have now added extensive new data to investigate the titanisation response of 42 strains across the Cryptococcus species complex, including multiple strains of every major lineage (Table 1)*

**Reviewer 2**

**1. Lines 89, 96: The use of the term “giant” cells here is unclear. Since one of the first two groups to identify titan cells referred to them as “giant” cells, this could be interpreted as another term for titans rather than large cells that are not yet conclusively titan.**

*We have now revised our manuscript to use ‘Titan’ consistently throughout to avoid confusion.*

**2. The data presented in figure 2 demonstrating that titan cells increase capsule size but do not appear to be actively growing is fascinating. Are these cells metabolically active? Could they represent a fungal phenomenon analogous to bacterial persister cells? Do titans have a long chronological lifespan compared to cells grown long-term in RPMI?**

*We now include new data demonstrating the metabolic activity and viability of Titan cells. We agree entirely with the reviewer that the potential role of Titan cells as fungal ‘persisters’ is extremely interesting and we hope that the in vitro method for generating them that we report here will prove useful in investigating this aspect in the near future.*

**3. Does pABA induce titanisation in C. neoformans?**

We now include new data (Supplementary Figure 1) showing that pABA moderately increases the (very low) rate of titanisation in C. neoformans.

**Reviewer 3**

**1. The ploidy study should be confirmed in more strains, not only R265.**

*We now extend our investigation of in vitro titan cell induction to a total of 42 strains, across all major lineages within both C. gattii and C. neoformans (Table 1 and Supplementary Figures 5 and 6)*

**2. The role of p-aminobenzoic acid should be further explored. They should validate some of the hypothesis from the discussion. Does p-aminobenzoic acid have any effect on other strains and species, such as C. neoformans?**

*We now include these data in Supplementary Figure 1, as described above.*

**3. It is not clear how this protocol compares to those described in 2018. Also, I think that the authors should take advantage of this protocol to provide insights on the molecular mechanisms that differentiate titan cell formation in C. gattii compared to C. neoformans. To increase the significance of the article, I would strongly recommend to perform an RNAseq experiment and look at global changes in gene transcription.**

*We respectfully suggest that a detailed molecular mechanistic study of titanisation lies beyond the scope of this paper. However, we have undertaken some limited gene expression analysis (shown in Figure 6) that indicates an interesting pattern of cell cycle regulation as a key factor behind titan cell formation.*

**4. Line 84, results. The authors state that the discovery that C. gattii forms titan cells in RPMI was observed during experiments in which the yeasts were cocultivated with alveolar macrophages. Was serum present in the initial experiment? It is surprising that the authors do not evaluate the effect of serum in their experiments, since this was one of the main inducing factors that induce in vitro titanisation in C. neoformans in two of the protocols described in 2018.**

*We tested both serum-containing and serum-free media in our assay; both are effective, so we focus on serum-free media for most experiments since that dramatically reduces the chemical complexity of the environment. We have now added more detail on this to the manuscript in order to expand on this point.*

**MINOR ISSUES**

**Reviewer 1**

**1. The critical role of titan cells for Cryptococcus neoformans to establish infection should be tempered.**

*We have now modified this text.*

**2. Line 33. The discovery of this protocol is probably less serendipitous than the first observations in mice and for C. neoformans in vitro protocols.**

*We have now changed this phrasing.*

**Reviewer 2**

1. The bars representing the median (? mean? not stated; should be) are very difficult to see in Figure 6H.

*All of the size distribution plots are shown as scatterplots with the mean, +/- standard deviation. We have now changed colours on some of the plots to make this clearer and have included a sentence explaining the error bars to the new methods section on Statistical Analysis.*

**Reviewer 3**

**1. Introduction should be rewritten. In particular, the authors should give more background, and summarize the findings of the article.**

*We have now extensively rewritten the introduction.*

**2. The authors should revise the English style, and make sure that past tense is always used when explaning the results. Furthermore, they should avoid the use of "giant" cells, since it can be confused with "giant" macrophages.**

*Now corrected*

**3. Statistics. The authors should include a specific statistics section in M&M.**

*Now added.*

**4. Furthermore, they have used ANOVA throughout the manuscript. However, this test can only be used when there is a normal gaussian distribution. And it seems that some of the size distribution are not normally distributed. They should assess the normality of all the samples (Shapiro-Wilk or Kolmogorov-Smirnov tests, and then evaluate whether a parametric (ANOVA) or non-parametric (i.e. Kruskal-Wallis) test should be applied.**

*We applied the Shapiro-Wilk test to all of our data and used either parametric (ANOVA) or non-parametric (Kruskal-Wallis or Mann-Whitney) tests accordingly, as described in the relevant figures.*

**5. The definition of titan cell and thresholds used is confusing. In some cases, they use a threshold of 10 um, and in other figure they use a threshold of 15 um. This should be unified to 10 um and the data recalculated in all the figures (i.e, figure 6). If the threshold defined by the authors for titan cells is 10 um (cell body diameter), why do they use filters of 15 um pore size?**

*We now use 10um throughout the manuscript. We used 15um pore filters (rather than 10um) in order to exclude small (non-titan) yeast cells with large capsules, that would otherwise exceed a 10um diameter.*

**6. It would be interesting to know if progeny from titan cells have a different chitin content compared to regular cells.**

*Although we have not characterised this in detail, there do not appear to be any fundamental differences in cell wall structure between titan ‘progeny’ and normal yeast cells.*

**7. I think that it is more correct to show the cell cycle and ploidy data (DAPI histograms) in lineal axis and not logarithmics, since the increse in DNA content is lineal.**

*The reviewer is of course correct that the ploidy increase is linear and not exponential. However, the fluorescence signal recorded by the flow cytometer is not a straightforward linear signal – for instance, the DAPI signal for wildtype control cells ranges across more than a log, even though the maximum variation in DNA content is two-fold. For this reason, we have retained the log scale for these histograms.*

**8. Figure 6. I would argue against the concept that C. gattii produces large haploid but non-titan cells. Titan cells should be defined by their size, since it is possible to obtain titan cells in different ways and through different mechanisms. I feel that from an imunological point of view, the main factor that determines immunological evasion is the size and not DNA content.**

*We think the reviewer is suggesting that the definitive feature of titan cells should be size only, regardless of ploidy. Whilst that is a valid point, the field as a whole has agreed on a definition that includes size, ploidy and cell wall modification and therefore we have stuck to the original nomenclature that does not classify haploid large cells as ‘titans’.*

**9. Figure 6G. The authors should show the size of cells grown in YPD too.**

*Now added (this is Figure 7a in the new submission)*

**10. Regarding cell density, it has been shown in C. neoformans that cell density and quorum sensing phenotypes depend on a short peptide (QSP1), and that this peptide inhibits in vitro titanisation in C. neoformans (reference 14 and 15). I think that the authors should investigate if this peptide or other QS molecules have a similar effect in TC formation in C. gattii.**

*We appreciate this excellent suggestion from the reviewer, which we have now undertaken. These new data (Figure 3b) indeed show that QSP1 is able to partially suppress titanisation at low density, indicating a key role for quorum sensing in this process.*

**11. Presentation of figure 5 is somehow confusing. I believe that it would be better to normalize the expression level in YPD to an arbitrary value of 1, and then present the rest of the data as fold-change compared to this condition.**

*We respectfully disagree. Since a key message of this figure is that cells cease budding and begin DNA reduplication during titanisation, we feel it is important for readers to be able to compare that pattern with the reverse pattern in YPD-grown cells. That comparison would be lost if we were to normalise the data to 1 in YPD-grown cells.*

**12. Table 2. Cryptococcus neoformans H99 strain is serotype A, VNI. This should be corrected.**

*Now corrected*

**13. Line 576. The authors state in the RNA isolation section that it was done in 400 uL of "RNAase". Do they mean "RNAeay buffer"?**

*A good spot – now corrected; thanks!*

**14. RT-qPCR experiments. It seems that the samples were not treated with DNAse to ensure that there is not interference in the RT_qPCR of contaminant DNA. If this step was omitted, they authors should have carried out a control sample in which the RNAs are equally treated, but without the addition of reverse transcriptase, to make sure that these samples are negative. I recommend to confirm this, since this step can be very tricky, and DNA contamination can occur.**

*We apologise for that oversight. Genomic DNA was indeed removed from the samples, but we omitted to specify that in the manuscript. We have now made that point clear.*

**15. Figure 4. It would be interesting to know what happens when titan cells recovered by filtration are incubated in fresh YPD and RPMI medium. Most probably, these cells are nutrient-depleted by day 2 of incubation, so it raises the issue of how do they behave when they are incubated again in a inducing and non-inducing medium.**

*We now include data examining the behaviour of Titan cells placed into fresh media, as well as characterising the progeny that they produce. These are described in Figures 4, 5 and Supplementary Figure 3.*

**16. RPMI vs DMEM experiments. The authors should give the exact reference of the media used, and in particular, which glucose concentration do they contain.**

*Now added. We in fact tried both low and high glucose conditions (Supplementary Figure 1), but saw no difference in titan cell formation between them.*

**17. Furthermore, the authors should ensure that during the whole experiments (even through day 7), there is not a change in pH, and that these two media have the same buffering strenght for C. gattii. Yeast can significantly alter the pH medium by the secretion of protons or ammonia, and I am not sure how these media can buffer in these conditions without the addition of an additional buffer.**

*We have now measured the pH of the different media we used, before and after induction, and include those data in Supplementary Figure 1.*

**18. Lines 358-363. The authors demonstrate that addition of iron inhibit titan cell development in C. gattii. This was also shown in C. neoformans (reference 14), so this should be highlighted.**

*Done*.
